# Trends in massive transfusion practice for trauma in Japan from 2011 to 2020: a nationwide inpatient database study

**DOI:** 10.1186/s40560-023-00685-0

**Published:** 2023-10-18

**Authors:** Hiroyuki Ohbe, Takashi Tagami, Akira Endo, Shigeki Miyata, Hiroki Matsui, Kiyohide Fushimi, Shigeki Kushimoto, Hideo Yasunaga

**Affiliations:** 1 Department of Clinical Epidemiology and Health Economics, School of Public Health, The University of Tokyo, 7-3-1 Hongo, Bunkyo-ku, Tokyo, 113-0033 Japan; 2 Division of Emergency and Critical Care Medicine, Tohoku University Graduate School of Medicine, Sendai, Japan; 3 Department of Emergency and Critical Care Medicine, Nippon Medical School Musashikosugi Hospital, Kawasaki, Kanagawa Japan; 4 Department of Acute Critical Care Medicine, Tsuchiura Kyodo General Hospital, Tsuchiura, Japan; 5 Central Blood Institute, Blood Service Headquarters, Japanese Red Cross Society, Tokyo, Japan; 6 Department of Health Policy and Informatics, Tokyo Medical and Dental University Graduate School, Tokyo, Japan

**Keywords:** Trauma, Massive transfusion, Transfusion ratio, Trend, Administrative database

## Abstract

**Background:**

Previous studies have reported conflicting results regarding fresh frozen plasma (FFP)-to-red blood cell (RBC) ratio and platelet-to-RBC ratio on outcomes for massive transfusion for trauma. Moreover, nationwide data on massive transfusion practices for trauma in the real-world clinical setting are scarce. This study aimed to examine the nationwide practice patterns and trends in massive transfusion for trauma in Japan using a national administrative, inpatient database.

**Method:**

We identified patients who underwent emergency hospitalization for trauma and received massive transfusion, defined as administration of at least 20 units of RBC within the first 2 days of admission, using the nationwide inpatient database, which covers approximately 90% of all tertiary emergency hospitals in Japan, between 2011 and 2020. Trends in the incidence and practice patterns of massive transfusion were described by calendar year. The association of practice patterns with mortality or adverse events was tested.

**Results:**

A total of 3,530,846 trauma hospitalizations were identified, of which 5247 (0.15%) received massive transfusion. A significant declining trend was observed in the incidence of massive transfusion in trauma hospitalizations from 0.24% in 2011 to 0.10% in 2020 (*P* for trend < 0.001). The FFP-to-RBC ratio rose significantly from 0.77 in 2011 to 1.08 in 2020 (*P* for trend < 0.001), while the platelet-to-RBC ratio remained virtually unchanged from 0.71 in 2011 to 0.78 in 2020 (*P* for trend 0.060). Massive transfusion with lower FFP-to-RBC (< 0.75) and platelets-to-RBC ratio (< 1.00) were associated with increased in-hospital mortality compared with those ≥ 1.00, while there were linear increases in adverse events with increasing FFP and platelets ratios.

**Conclusions:**

This study demonstrated a declining trend in the incidence and a rise in higher FFP-to-RBC ratios in massive transfusion in association with patient outcomes for trauma from 2011 to 2020 in Japan.

**Supplementary Information:**

The online version contains supplementary material available at 10.1186/s40560-023-00685-0.

## Introduction

Massive hemorrhage is a significant cause of death due to trauma [1]. Over the past two decades, the treatment strategy for massive hemorrhage has undergone a paradigm shift from aggressive definitive surgery and volume resuscitation with crystalloids to damage control surgery, early administration of blood components for prevention of coagulopathy, and restrictive crystalloid replacement [2]. Patients who have experienced severe trauma with hemorrhagic shock often require massive transfusion, and the massive transfusion protocol has become a pillar of damage control resuscitation in recent trauma guidelines [3, 4].

While various definitions exist for massive transfusion, it is most commonly defined as transfusion of ≥ 10 units of packed red blood cells (RBCs) within 24 h of trauma [5, 6]. The massive transfusion protocol is a set of guidelines and procedures implemented in hospitals to facilitate the rapid delivery of blood products in a predefined ratio of RBC, fresh frozen plasma (FFP), and platelets [3–6]. Current guidelines recommend a massive transfusion protocol with an FFP-to-RBC ratio of at least 1:2 [3, 4]. The PROPPR trial, which was published in February 2015, showed that a massive transfusion protocol with an FFP, platelet, and RBC ratio of 1:1:1 enabled quicker hemostasis and decreased the frequency of 24-h mortality due to exsanguination, but did not reduce the 30-day mortality, compared to a ratio of 1:1:2 [7]. Several multi-center, observational studies support a higher plasma or platelet-to-RBC ratio for massive transfusion [8–13], while others have failed to demonstrate any benefit of the same [14–17]. Currently, there is no established practice and protocol for massive transfusion, and the manner in which massive transfusion is currently implemented for trauma in real-world clinical settings remains unclear.

According to a previous nationwide hospital survey conducted in 2016 in Japan, 31 of 82 tertiary emergency medical facilities devised a massive transfusion protocol: the majority of these institutions established a target ratio of FFP-to-RBC ratio of 1:1 [18]. However, only 30% (*n* = 82/279) of the tertiary emergency medical facilities participated in the study and the massive transfusion practice and outcomes remain unknown.

Therefore, this study aimed to examine the nationwide practice patterns and trends in massive transfusion for patients with trauma in Japan using a nationwide administrative, inpatient database.

## Methods

### Study design and data source

This observational study was conducted using the Diagnosis Procedure Combination database, a nationwide Japanese administrative inpatient database. This study followed the Strengthening the Reporting of Observational Studies in Epidemiology (STROBE) reporting guideline.

The database contains discharge summaries and administrative claims from more than 1500 acute-care voluntarily participating hospitals and data from approximately 50% of all acute hospitals and 90% of all tertiary emergency hospitals in Japan [19]. It includes the following patient-level data for all hospitalizations: demographic characteristics; primary diagnoses, comorbidities, and complications recorded with the International Classification of Diseases, 10th Revision (ICD-10) codes; daily procedures; daily drug administrations; daily blood product administrations; and admission and discharge status. A previous study that validated this database showed high specificity and moderate sensitivity for the recorded diagnoses and high specificity and sensitivity for the recorded procedures, although the trauma-specific diagnostic codes were not examined [20].

### Study population

We identified patients who were hospitalized for trauma (ICD-10 codes: S00–T14 for the primary diagnosis) on an emergency basis by ambulance or walk-in between January 1, 2011, and December 31, 2020. We enrolled patients who received massive transfusion, defined as administration of at least 20 units of RBC in Japan (equivalent to 10 units of RBC in the USA or UK) within the first 2 days of admission. One unit of packed RBCs is equal to approximately 140 mL in Japan, 250–350 mL in the USA, and 280 mL in the UK.

### Data collection

Data on the following characteristics were collected from the database: calendar year of admission, i.e., 2011 to 2020; hospital characteristics (tertiary emergency or teaching hospital); age, sex, and body mass index at admission; Japan Coma Scale at admission [21], Charlson comorbidity index score [22]; ambulance use, regions to which injury was sustained, ICD-10-based injury severity score [23]; and treatments administered within the first 2 days of admission. The ICD-10 codes for the injured regions are listed in Additional file 1: Table S1. The severity of trauma was assessed using a validated ICD-10-based injury severity score [23]. Unavailable values for the body mass index at admission were treated as a missing category.

### Outcomes

The study outcomes were in-hospital mortality and incidence of adverse events. Adverse events were defined as a composite of cardiac failure, respiratory failure, hepatic failure, renal failure, sepsis, thrombosis, transfusion transmitted viral infections, allergic/anaphylactic reactions, hemolytic transfusion reaction, and volume overload (besides the above) based on the definition of transfusion-related adverse events in previous studies [24, 25]. The ICD-10 codes used to identify adverse events are shown in Additional file 1: Table S2. Data on death in the emergency room, death within 24 h of admission, duration of hospitalization, and hospitalization costs were also collected.

### Statistical analysis

The trends in the incidence and practice patterns of massive transfusion for patients with trauma were described by calendar year at admission from 2011 to 2020, and analyzed using the Cochran–Armitage trend test for binary variables and Jonckheere–Terpstra trend test for continuous variables [26]. The incidence of massive transfusion was calculated using the “number of hospitalizations for trauma that received at least 20 units of RBC within the first 2 days of admission” as the numerator and “the number of hospitalizations for (i) all trauma; (ii) trauma in a tertiary emergency hospital; (iii) trauma requiring admission to the intensive care unit or high-dependency care unit; and (iv) trauma requiring at least one unit of RBCs” as the denominator. The trends in massive transfusion-related procedures within the first 2 days of admission and consumption rate of blood products during hospitalization for patients who received massive transfusion from among the entire trauma population were also examined in a similar manner.

Restricted cubic spline analyses were performed to assess the non-linear association between the outcomes and transfusion ratios (FFP-to-RBC ratio and platelet-to-RBC ratio) [27]. Five transfusion ratio points (0.50, 0.75, 1.00, 1.25, and 1.50) were denoted as the knots. We fitted generalized estimating equations to the restricted cubic spline analyses with individual hospitals as the cluster and calculated the adjusted odds ratios and their 95% confidence intervals for each transfusion ratio relative to the reference point of 1.00. In a different analysis, transfusion ratios were categorized into four groups: 0.75 or less; 0.75 to 1.00; 1.00 to 1.25; over 1.25, and generalized estimating equations with individual hospitals as the cluster were created to assess the association between the four transfusion ratio categories and the outcomes, using 0.75 to 1.00 as the reference category. All adjusted analyses included the calendar year at admission, hospital characteristics, age, sex, body mass index at admission, Japan Coma Scale at admission, Charlson comorbidity index, ambulance use, injured regions, and ICD-10-based injury severity score as covariates.

Sensitivity analyses were performed by excluding patients who died in the emergency room in order to reduce survivor bias, because patients requiring massive transfusion often die during the early hours of admission before receiving substantial quantities of FFP or platelets [15, 28, 29]. Furthermore, post hoc sensitivity analyses were performed (i) by altering the definition of massive transfusion to patients who received at least 20 units of RBC on the day of admission; (ii) by altering the definition of massive transfusion to patients who received at least 60 total units of RBC, FFP, and platelets within the first 2 days of admission; (iii) by restricting the sample to patients admitted to tertiary emergency centers; and (iv) by restricting the sample to patients who were admitted to hospitals that had continuously provided data to the database from 2011 to 2020.

All analyses were performed using Stata/SE 17.0 software (StataCorp, College Station, TX, USA). Continuous variables were presented as means and standard deviations or medians and interquartile ranges as appropriate, and categorical variables were presented as numbers and percentages. All reported *P*-values were two-sided, and *P*-values < 0.05 were considered statistically significant.

## Results

A total of 3,530,846 hospitalizations for trauma were identified from 1811 hospitals during the 10-year study period (Table 1). Of these, 5247 (0.15%) patients received massive transfusion. The number of hospitalizations for trauma did not change significantly (*P* for trend 0.089), but the number of hospitalizations for trauma with massive transfusion showed a significant decline (*P* for trend 0.040) over the 10-year study period. The incidence of massive transfusion for all traumas exhibited a significant declining trend, ranging from 0.24% in 2011 to 0.10% in 2020 (*P* for trend < 0.001). Similar decreasing trends were observed when the incidence of massive transfusion was calculated using different denominators. The overall consumption rates of blood products for massive transfusion patients in all trauma patients were 8.3%, 26.0%, and 19.2% for RBC, FFP, and platelets, respectively. The consumption rate of RBCs decreased significantly from 11.6% in 2011 to 5.8% in 2020 (*P* for trend < 0.001), while that of FFP and platelets did not change significantly.

**Table 1:** Trends in the incidence and blood product consumption for trauma requiring massive transfusion

	Formula	Total	Calendar year										*P* for
			2011	2012	2013	2014	2015	2016	2017	2018	2019	2020	trend
Number of hospitals, n	–	1811	1114	1185	1187	1273	1353	1387	1331	1318	1229	1211	–
Hospitalizations for trauma, n	A	3,530,846	226,202	309,254	326,474	363,141	379,347	405,447	404,825	393,090	369,497	353,569	0.089
In a tertiary emergency hospital	B	1,225,865	83,687	108,442	118,070	127,429	129,635	136,136	137,943	134,157	130,102	120,264	0.040
With at least one unit of RBCs	C	182,242	12,140	15,459	15,875	17,890	19,330	20,749	20,847	20,167	19,769	20,016	0.010
With ≥ 10 units of RBCs	D	17,859	1662	1788	1913	1969	1871	1971	1855	1743	1651	1436	0.24
With ≥ 20 units of RBCs	E	5247	532	524	585	594	571	586	517	501	473	364	0.040
Number of hospitals performing MT, n	–	486	188	213	211	210	203	214	204	197	182	161	–
Hospital volume of MT, median (IQR)	–	2 (1,3)	2 (1,3)	2 (1,3)	2 (1,3)	2 (1,3)	2 (1,4)	2 (1,3)	2 (1,3)	2 (1,3)	2 (1,3)	2 (1,3)	–
Incidence of MT*, %													
Denominator													
All trauma	E/A	0.15	0.24	0.17	0.18	0.16	0.15	0.14	0.13	0.13	0.13	0.10	< 0.001
In a tertiary emergency hospital	E/B	0.43	0.64	0.48	0.50	0.47	0.44	0.43	0.37	0.37	0.36	0.30	< 0.001
Requiring at least one unit of RBCs	E/C	2.88	4.38	3.39	3.69	3.32	2.95	2.82	2.48	2.48	2.39	1.82	< 0.001
*Consumption of blood product*^*†*^*, unit*													
For all trauma patients													
RBC	F	2,481,513	177,676	225,663	230,852	254,098	267,562	279,670	277,609	265,882	254,770	247,733	0.13
FFP	G	713,513	52,486	66,888	71,158	71,241	75,611	78,117	79,282	77,552	74,512	66,666	0.13
Platelets	H	904,451	64,491	85,824	92,324	98,878	100,032	99,736	99,205	94,071	91,106	78,784	0.79
For trauma requiring MT													
RBC	I	205,134	20,538	19,941	22,228	22,458	22,551	22,919	21,063	19,892	19,201	14,343	0.25
FFP	J	185,281	16,490	16,156	18,703	19,203	20,801	21,180	19,614	19,321	19,366	14,447	0.42
Platelets	K	173,250	14,674	15,660	18,589	18,956	20,000	20,118	19,125	16,897	17,296	11,935	0.79
Consumption rate for MT^‡^, %													
RBC	I/F	8.3	11.6	8.8	9.6	8.8	8.4	8.2	7.6	7.5	7.5	5.8	< 0.001
FFP	J/G	26.0	31.4	24.2	26.3	27.0	27.5	27.1	24.7	24.9	26.0	21.7	0.18
Platelets	K/H	19.2	22.8	18.2	20.1	19.2	20.0	20.2	19.3	18.0	19.0	15.1	0.060

RBC: red blood cell; MT: massive transfusion; IQR: interquartile range; FFP: fresh frozen plasma

*While calculating the incidence of massive transfusion, the numerator was the total number of hospitalizations for trauma requiring massive transfusion defined as the administration of at least 20 units of red blood cells within the first two days of admission and the denominator was the total number of hospitalizations for all trauma, trauma in a tertiary emergency hospital, or trauma requiring at least one unit of RBCs

^†^The consumption of blood products was calculated using the sum of those used during hospitalization

^‡^While calculating the consumption rate for massive transfusion, the numerator was the total number of blood products administered for patients with trauma requiring massive transfusion; and the denominator was the total number of blood products administered to all patients with trauma

The mean age of the 5247 patients who received massive transfusion was 56.9 years and 64.0% were men (Table 2). Overall, 50.1% of patients experienced abdominal and pelvic injury, 18.0% had thoracic injury and 16.6% experienced head injury. Surgery was performed under general anesthesia for 69.8% of patients within 2 days of admission. The overall in-hospital mortality and frequency of adverse events were 39.5% and 20.6%, respectively. The results for each of the complications are presented in Additional file 1: Table S3. The trends in the characteristics and outcomes are presented in Additional file 1: Table S4. During the study period, a significant rising trend was observed in the proportion of patients with a body mass index of 25.0–29.9, coma at admission, higher ICD-10-based injury severity score, interventional radiology, vasopressors, and adverse events. The in-hospital mortality did not change significantly from 43.4% in 2011 to 41.2% in 2020 during the study period (*P* for trend 0.96). The frequency of death in the emergency or operating room and death within 24 h also did not change significantly during the study period.

**Table 2:** Characteristics and outcomes patients with trauma requiring massive transfusion

	Overall
	(*n* = 5247)
Hospital characteristics	
Tertiary emergency hospital, *n* (%)	4650 (88.6)
Teaching hospital, *n* (%)	5237 (99.8)
Age, years, mean (SD)	56.9 (22.2)
Male, *n* (%)	3358 (64.0)
Body mass index at admission, kg/m^2^, *n* (%)	
< 18.5	513 (9.8)
18.5–24.9	2602 (49.6)
25.0–29.9	787 (15.0)
≥ 30.0	176 (3.4)
Missing	1169 (22.3)
Japan Coma Scale at admission, *n* (%)	
Alert	1065 (20.3)
Confusion	1007 (19.2)
Somnolence	818 (15.6)
Coma	2357 (44.9)
Charlson comorbidity index, mean (SD)	0.2 (0.6)
Ambulance use, *n* (%)	4991 (95.1)
Injured region, *n* (%)	
Head	869 (16.6)
Neck	102 (1.9)
Thorax	944 (18.0)
Abdomen and pelvis	2629 (50.1)
Extremities	596 (11.4)
Multiple	1260 (24.0)
ICD-10-based injury severity score, mean (SD)	2.6 (2.1)
Treatment within 2 days of admission, *n* (%)	
Intensive care unit admission	4012 (76.5)
High-dependency care unit admission	1198 (22.8)
Surgery with general anesthesia	3665 (69.8)
Interventional radiology	2206 (42.0)
Mechanical ventilation	4132 (78.7)
Vasopressor administration	4622 (88.1)
Outcomes	
In-hospital mortality, *n* (%)	2073 (39.5)
Adverse events, *n* (%)	1080 (20.6)
Death in the emergency or operating room, *n* (%)	157 (3.0)
Death within 24 h, *n* (%)	1032 (19.7)
Duration of hospitalization, days, mean (SD)	48.8 (67.0)
Hospitalization cost, 1000 yen, mean (SD)	5631 (4145)

SD: standard deviation; ICD-10: International Classification of Diseases, 10th Revision

**Fig. 1:**
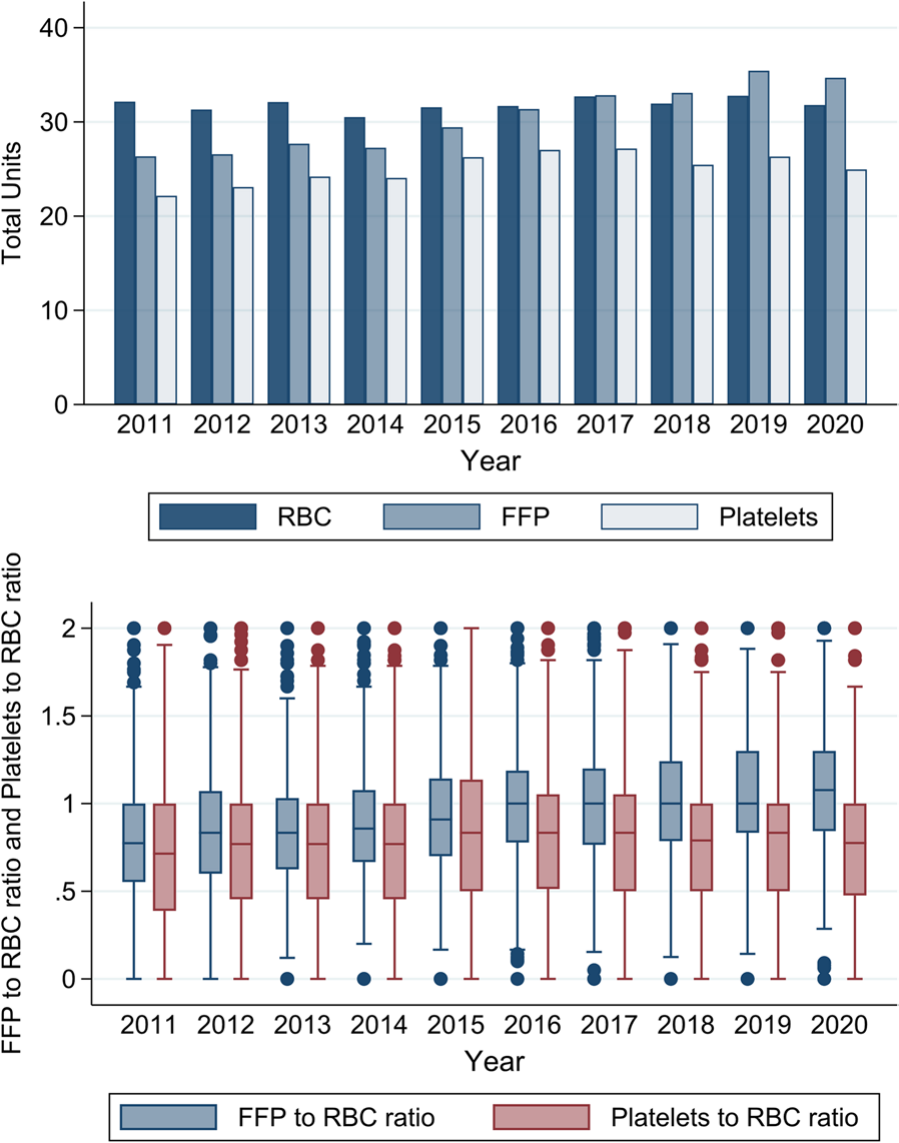
Trends in the mean total units of RBC, FFP, platelets, FFP-to-RBC ratio, and platelet-to-RBC ratio used for massive transfusion in Japan from 2011 to 2020. The mean total units of RBCs, FFP, platelets were calculated using the units administered within the first 2 days of hospitalization. In the box plot, the horizontal line indicates the median, and the upper-most and lower-most borders of the box denote the 75th and 25th percentiles, respectively. The whiskers above and below the box mark the 90th and 10th percentiles, respectively. The points beyond the whiskers are outliers beyond the 90th percentile. RBC: red blood cell; FFP: fresh frozen plasma

The trends in massive transfusion within the first 2 days of admission are shown in Table 3 and Fig. 1. The trends in the mean total units of RBC transfused did not change (32.1 units in 2011 and 31.8 units in 2020, *P* for trend 0.33), while there were significant rise trends in those of FFP (26.4 units and 34.7 units, *P* for trend < 0.001) and platelets (22.2 units and 25.0 units, *P* for trend 0.040). The FFP-to-RBC ratio rose significantly from 0.77 in 2011 to 1.08 in 2020 (*P* for trend < 0.001), while the platelet-to-RBC ratio did not change significantly from 0.71 in 2011 to 0.78 in 2020 (*P* for trend 0.060). The use of tranexamic acid and fibrinogen concentrate increased significantly during the study period.

**Table 3:** Trends in the products transfused during massive transfusion within the first 2 days of admission

Procedures*	Calendar year										*P* for trend
	2011	2012	2013	2014	2015	2016	2017	2018	2019	2020	
RBCs, unit, mean	32.1	31.3	32.1	30.5	31.6	31.7	32.7	31.9	32.8	31.8	0.33
FFP, unit, mean	26.4	26.6	27.7	27.3	29.4	31.4	32.8	33.1	35.4	34.7	< 0.001
Platelets, unit, mean	22.2	23.1	24.2	24.1	26.3	27.0	27.2	25.5	26.3	25.0	0.040
FFP-to-RBC ratio, median	0.77	0.83	0.83	0.86	0.90	1.00	1.00	1.00	1.00	1.08	< 0.001
Platelet-to-RBC ratio, median	0.71	0.77	0.77	0.77	0.83	0.83	0.83	0.79	0.83	0.78	0.060
Crystalloid, L, median	13.7	13.8	13.1	13.6	13.2	13.2	13.1	13.3	12.2	12.5	0.031
Tranexamic acid, %	44.0	48.7	52.3	54.2	62.2	62.6	64.8	67.5	65.3	73.6	< 0.001
Fibrinogen concentrate, %	1.3	2.7	5.3	3.4	4.4	4.1	5.4	5.6	8.2	12.1	< 0.001
Recombinant factor VIIa, %	0.6	1.7	1.7	1.2	0.7	0.9	0.8	0.4	0.6	0.8	0.087
Cryoprecipitate, %	0.0	0.0	0.0	0.0	0.0	0.0	0.0	0.0	0.0	10.4	< 0.001

RBC: red blood cell; IQR: interquartile range; FFP: fresh frozen plasma

^*^Massive transfusion-related procedures were evaluated within the first 2 days of admission

**Fig. 2:**
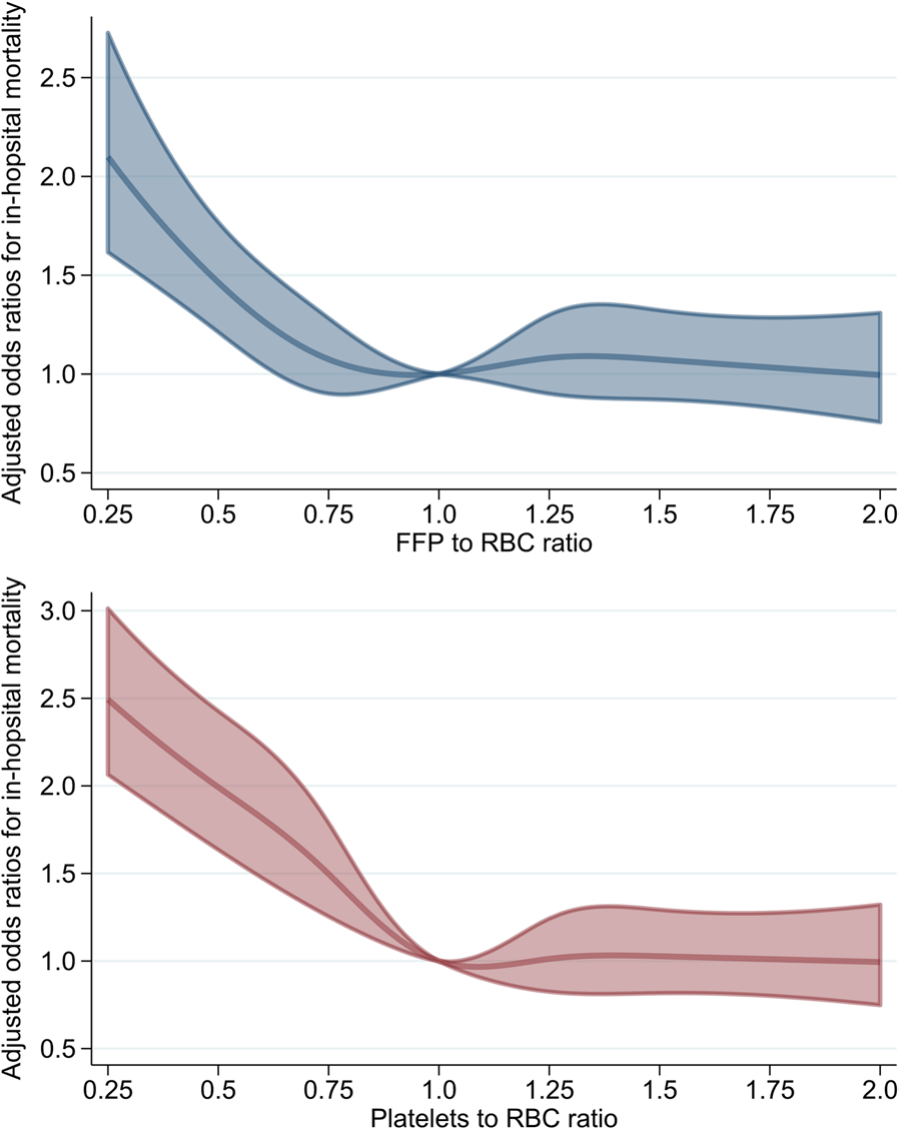
Non-linear associations between the FFP-to-RBC ratio or platelet-to-RBC ratio and in-hospital mortality in the restricted cubic spline analyses. The model was adjusted for calendar year at admission; hospital characteristics; age, sex, and body mass index at admission; Japan Coma Scale at admission; Charlson comorbidity index; ambulance use; injured regions; and the ICD-10-based injury severity score as covariates. Five transfusion ratio points (0.50, 0.75, 1.00, 1.25, and 1.50) were denoted as the knots and 1.00 was designated as the reference value. The area region represents 95% confidence intervals for the estimated adjusted odds ratios. FFP: fresh frozen plasma; RBC: red blood cell; ICD-10: International Classification of Diseases, 10th Revision

**Fig. 3:**
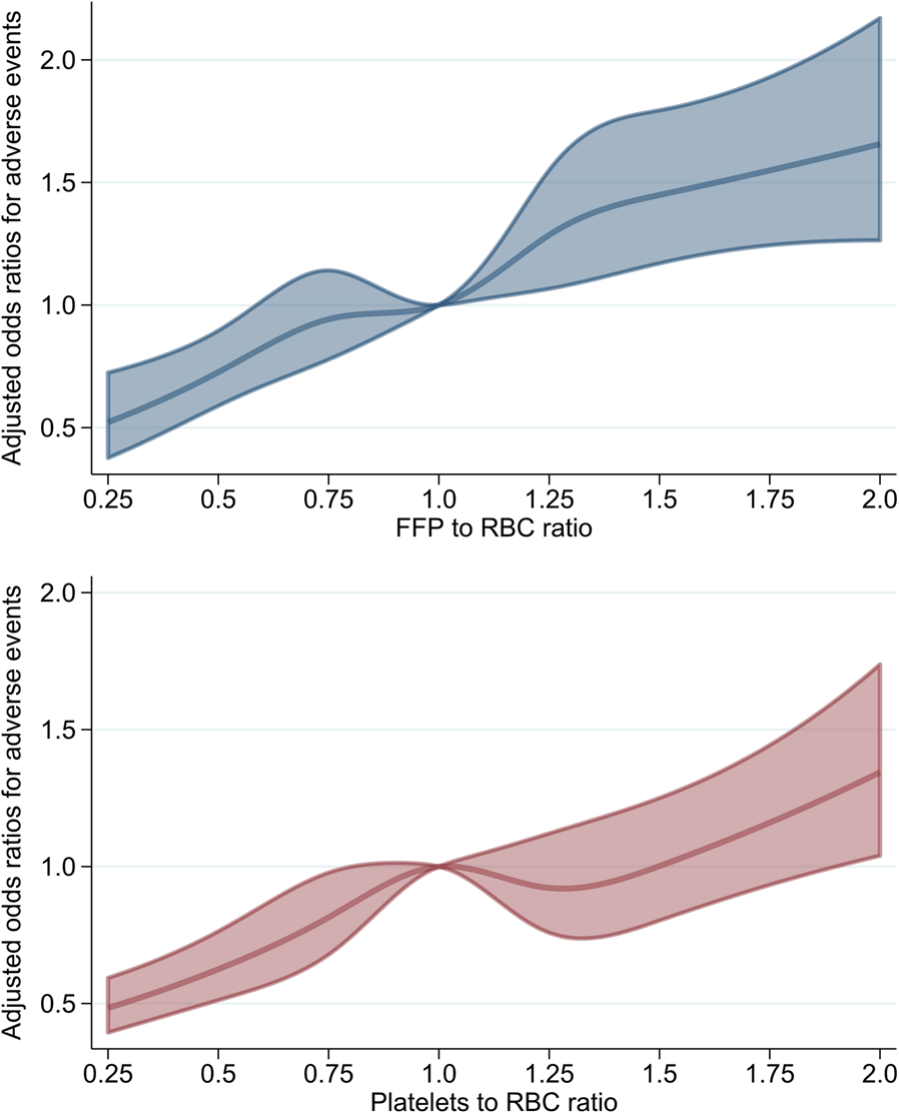
Non-linear associations between the FFP-to-RBC ratio or platelet-to-RBC ratio and adverse events in the restricted cubic spline analyses. The model was adjusted for calendar year at admission; hospital characteristics; age, sex, and body mass index at admission; Japan Coma Scale at admission; Charlson comorbidity index score; ambulance use; injured regions; and the ICD-10-based injury severity score as covariates. Five transfusion ratio points (0.50, 0.75, 1.00, 1.25, and 1.50) were denoted as the knots and 1.00 was denoted as the reference. The area region represents 95% confidence intervals for the estimated adjusted odds ratios. FFP: fresh frozen plasma; RBC: red blood cell; ICD-10: International Classification of Diseases, 10th Revision

Restricted cubic spline analysis for in-hospital mortality showed higher adjusted odds ratios for FFP-to-RBC ratio < 0.75 and platelet-to-RBC ratio < 1.00, but no change in transfusion ratios > 1.00 (Fig. 2). Restricted cubic spline analysis for adverse events showed a linear increase in the adjusted odds ratios for the FFP-to-RBC ratio and platelet-to-RBC ratio (Fig. 3). Generalized estimating equations with the four transfusion ratio categories yielded similar findings for adverse events (Table 4). The results of sensitivity analyses conducted by excluding 157 patients who died in the emergency room were similar to those of the main analyses (Additional file 1: Tables S5, S6 and Figs. S1, S2). The results of four post hoc sensitivity analyses were similar to those of the main analyses (Additional file 1: Tables S7–S14 and Figs. S3–S10).

**Table 4:** Results of generalized estimating equations devised to assess the association between the four transfusion ratio categories and the outcomes

	In-hospital mortality			Adverse events		
	Number (%)	Adjusted odds ratio (95% CI)	*P* value	Number (%)	Adjusted odds ratio (95% CI)	*P* value
FFP-to-RBC ratio						
− 0.75	720/1637 (44.0)	1.27 (1.08–1.50)	0.005	259/1637 (15.8)	0.79 (0.66–0.95)	0.011
0.75–1.00	646/1731 (37.3)	Reference	–	347/1731 (20.1)	Reference	–
1.00–1.25	340/959 (35.5)	0.96 (0.80–1.16)	0.67	225/959 (23.5)	1.21 (1.00–1.46)	0.056
1.25–	367/920 (39.9)	1.09 (0.90–1.32)	0.39	249/920 (27.1)	1.49 (1.23–1.82)	< 0.001
Platelet-to-RBC ratio						
− 0.75	1162/2408 (48.3)	1.93 (1.65–2.26)	< 0.001	375/2408 (15.6)	0.62 (0.52–0.73)	< 0.001
0.75–1.00	521/1578 (33.0)	Reference	–	374/1578 (23.7)	Reference	–
1.00–1.25	165/495 (33.3)	1.14 (0.90–1.45)	0.29	127/495 (25.7)	1.12 (0.88–1.41)	0.35
1.25–	225/766 (29.4)	0.84 (0.68–1.04)	0.11	204/766 (26.6)	1.17 (0.95–1.43)	0.13

The model was adjusted for calendar year at admission; hospital characteristics; age, sex, and body mass index at admission; Japan Coma Scale at admission; Charlson comorbidity index score; ambulance use; injured regions; and ICD-10-based injury severity score as covariates

CI: confidence interval; RBC: red blood cell; FFP: fresh frozen plasma; ICD-10: International Classification of Diseases, 10th Revision

## Discussion

This study investigated the nationwide real-word trends in massive transfusion practice and yielded three salient findings. First, the incidence of massive transfusion decreased from 2011 to 2020, even when the denominator was trauma in a tertiary emergency hospital or trauma requiring blood transfusion. Second, the FFP-to-RBC ratio increased, while the platelet-to-RBC ratio did not change from 2011 to 2020 among patients who received massive transfusion. Third, massive transfusion within the first 2 days of admission with a lower FFP-to-RBC ratio of < 0.75 and lower platelet-to-RBC ratio of < 1.00 was associated with increased in-hospital mortality compared to ratios ≥ 1.00, while there was a linear increase in the frequency of adverse events with the increase in the FFP and platelet transfusion ratios.

Previous studies have shown that the incidence of trauma patients requiring massive transfusion ranged between 5 and 25%, depending on the definition of massive transfusion and study population [7, 30, 31]. The incidence of massive transfusion in this study was relatively lower than that reported by previous studies, and remained consistent even when the denominators of incidence were changed. A previous study conducted at a level 1 trauma center in the USA showed that patients with trauma requiring massive transfusion consumed 71% of all RBCs transfused to all trauma patients [32]; thus, the 8.3% RBC consumption rate in this study is relatively low. Considering that 88.6% of patients who required massive transfusion were admitted to tertiary emergency hospitals and that the database includes 50% of all acute hospitals and 90% of all tertiary emergency hospitals in Japan, the national annual incidence of massive transfusion for trauma can be estimated to be approximately 400 cases by 2021. These results may indicate that massive transfusion practice has a small impact on recent trauma care due to the small number of severe trauma cases in Japan. This may be attributed to the mechanism of traumatic injury in Japan, which is less severe than that in the USA, owing to the higher proportion of self-inflicted injuries, lower proportion of assaults, and lower proportion of penetrating injuries [33]. Our study also revealed a declining trend in the frequency of patients with trauma requiring massive transfusion, which can be explained by two hypotheses. First, the incidence of serious trauma itself is declining due to improved vehicle safety and stricter control of drink-driving in Japan. Second, increasing awareness of the importance of early and aggressive administration of FFP and platelets has led to earlier hemostasis and a reduction in total blood transfusion, resulting in fewer cases of massive transfusion [9, 10].

The present nationwide Japanese study showed that median FFP-to-RBC ratio increased gradually, while the platelet-to-RBC ratio was virtually unchanged. Thawed plasma is not available in Japan, and FFP must be used within 24 h of thawing. Moreover, in contrast to the situation in the USA, platelets are not always stocked in hospitals, and need to be transported from blood centers on demand (which usually takes approximately 30–60 min), hindering the use of higher FFP and platelet-to-RBC ratios for massive transfusion in the early stage of trauma. Despite these difficulties, the use of higher FFP-to-RBC ratio appears to have accelerated from 2015, possibly reflecting the clinical impact of the PROPPR trial published in February 2015. Interestingly, the platelet-to-RBC ratio in this study remained virtually unchanged after publication of the PROPPR trial. The relatively low platelet-to-RBC ratio should be a target for improving patient outcomes in Japan in the future.

The results of the adjusted analyses between the outcomes and transfusion ratios in this study supported the recent guideline recommendations of a 1:1:1 ratio for FFP, platelets, and RBCs for patients requiring massive transfusion [3, 4]. The results of this study were similar to those of several multi-center observational studies that showed a decrease in mortality with a higher plasma or platelet-to-RBC ratio for massive transfusion [8–13], and differed from the results of other studies that did not show any benefit [14–17]. Especially, it is not generally recognized that transfusions of FFP and platelets in a ratio of > 1:1 may be associated with worse outcomes; this finding could be considered in clinical practice and future research. Several clinicians agree that early and aggressive FFP and platelet transfusion can reduce mortality by preventing and immediately correcting coagulopathy [34, 35]. However, FFP and platelet transfusions are not free of risk; increased exposure to transfusions may increase the incidence of transfusion-related complications [36]. Considering that the transfusion ratios of FFP and platelet exhibited an S-shaped relationship with in-hospital mortality and a linear relationship with adverse events, excessive transfusions of FFP and platelets in a transfusion ratio of > 1:1 should be discouraged. But our results should be interpreted with caution. Survivor bias has been implicated as a confounder in previous analyses of transfusion ratios [15, 28, 29], and our study may not be exempt from its influence. Since the use of fibrinogen concentrate or cryoprecipitate has increased tremendously in recent years, further research on an optimal transfusion strategy that incorporated new blood products is needed.

This study has several limitations. First, the database did not contain detailed information on the patients’ physiological status, especially the hemoglobin level and blood pressure, which may have introduced bias. However, during the 10-year study period, the recommendations of the relevant Japanese societies for the target hemoglobin levels for RBC transfusion in patients with trauma have not changed [37, 38], and neither has consensus evidence emerged on the target blood pressure levels. Second, the use of all blood products, drugs, and procedures was recorded only on a daily basis, rather than by minute or hour. Therefore, massive transfusion was defined with respect to RBC administration during the first 2 days of admission, and not in the initial 24-h period. Furthermore, this study did not assess the massive transfusion protocol. Third, because this study was not based on a clinical trial, a causative relationship between the outcomes and transfusion ratios could not be inferred. Fourth, information on the mechanism of injury (blunt or penetrating) was not available in the database. However, in Japan, approximately 96% of patients with severe trauma sustain blunt injury according to a nationwide trauma registry; therefore, the present results may be applicable to many developed countries where blunt trauma is also the main mechanism of injury [33]. Fifth, in Japan, the use of fibrinogen concentrate for massive trauma-induced hemorrhage is not covered by national health insurance, while cryoprecipitate has been covered for massive trauma-induced hemorrhage since April 1, 2020; therefore, its use was underestimated in the current analysis.

## Conclusions

This study demonstrated a decreasing trend in the frequency of massive transfusion for patients with trauma and a rise in higher FFP-to-RBC ratios for massive transfusions within the first 2 days of admission from 2011 to 2020 in Japan. Similar studies are needed to capture the real-world practice patterns of massive transfusion for trauma in a nationwide clinical setting.

### Supplementary Information

**Additional file 1: Table S1.** ICD-10 codes for injured regions. Table S2. ICD-10 codes for adverse events. **Table S3.** Results for each of the complications. **Table S4.** Trends in the characteristics and outcomes of trauma patients requiring massive transfusion. **Table S5.** Trends in the incidence, blood products transfused, and outcomes of the sensitivity analysis by excluding 157 patients who died in the emergency room (*N* = 5090). **Table S6.** Results of the sensitivity analysis with generalized estimating equations to assess the association between the four transfusion ratio categories and outcomes conducted by excluding 157 patients who died in the emergency room (*N* = 5090). **Table S7.** Trends in the incidence, blood products transfused, and outcomes of the post hoc sensitivity analysis by altering the definition of massive transfusion to patients who received at least 20 units of RBC on the day of admission (*N* = 3238). **Table S8.** Results of the sensitivity analysis with generalized estimating equations to assess the association between the four transfusion ratio categories and outcomes conducted by altering the definition of massive transfusion to patients who received at least 20 units of RBC on the day of admission (*N* = 3238). **Table S9.** Trends in the incidence, blood products transfused, and outcomes of the post hoc sensitivity analysis by altering the definition of massive transfusion to patients who received at least 60 total units of RBC, FFP, and platelets within the first 2 days of admission (*N* = 4624). **Table S10.** Results of the sensitivity analysis with generalized estimating equations to assess the association between the four transfusion ratio categories and outcomes conducted by altering the definition of massive transfusion to patients who received at least 60 total units of RBC, FFP, and platelets within the first 2 days of admission (*N* = 4624). **Table S11.** Trends in the incidence, blood products transfused, and outcomes of the post hoc sensitivity analysis by restricting the sample to patients admitted to the tertiary emergency centers (*N* = 4650). **Table S12.** Results of the sensitivity analysis with generalized estimating equations to assess the association between the four transfusion ratio categories and outcomes conducted by restricting the sample to patients admitted to the tertiary emergency centers (*N* = 4650). **Table S13.** Trends in the incidence, blood products transfused, and outcomes of the post hoc sensitivity analysis by restricting the sample to patients who were admitted to hospitals that had continuously provided data to the database from 2011 to 2020 (*N* = 3783). **Table S14.** Results of the sensitivity analysis with generalized estimating equations to assess the association between the four transfusion ratio categories and outcomes conducted by restricting the sample to patients who were admitted to hospitals that had continuously provided data to the database from 2011 to 2020 (*N* = 3783). **Figure S1.** Non-linear associations between the FFP to RBC ratio or platelet to RBC ratio and in-hospital mortality revealed by sensitivity analyses with restricted cubic spline analysis conducted by excluding 157 patients who died in the emergency room. **Figure S2.** Non-linear associations between the FFP to RBC ratio or platelet to RBC ratio and adverse events in the sensitivity analyses with restricted cubic spline analysis conducted by excluding 157 patients who died in the emergency room. **Figure S3.** Non-linear associations between the FFP to RBC ratio or platelet to RBC ratio and in-hospital mortality revealed by sensitivity analyses with restricted cubic spline analysis conducted by altering the definition of massive transfusion to patients who received at least 20 units of RBC on the day of admission. **Figure S4.** Non-linear associations between the FFP to RBC ratio or platelet to RBC ratio and adverse events in the sensitivity analyses with restricted cubic spline analysis conducted by altering the definition of massive transfusion to patients who received at least 20 units of RBC on the day of admission. **Figure S5.** Non-linear associations between the FFP to RBC ratio or platelet to RBC ratio and in-hospital mortality revealed by sensitivity analyses with restricted cubic spline analysis conducted by altering the definition of massive transfusion to patients who received at least 60 total units of RBC, FFP, and platelets within the first 2 days of admission. **Figure S6.** Non-linear associations between the FFP to RBC ratio or platelet to RBC ratio and adverse events in the sensitivity analyses with restricted cubic spline analysis conducted by altering the definition of massive transfusion to patients who received at least 60 total units of RBC, FFP, and platelets within the first 2 days of admission. **Figure S7.** Non-linear associations between the FFP to RBC ratio or platelet to RBC ratio and in-hospital mortality revealed by sensitivity analyses with restricted cubic spline analysis conducted by restricting the sample to patients admitted to the tertiary emergency centers. **Figure S8** Non-linear associations between the FFP to RBC ratio or platelet to RBC ratio and adverse events in the sensitivity analyses with restricted cubic spline analysis conducted by restricting the restricting the sample to patients admitted to the tertiary emergency centers. **Figure S9.** Non-linear associations between the FFP to RBC ratio or platelet to RBC ratio and in-hospital mortality revealed by sensitivity analyses with restricted cubic spline analysis conducted by restricting the sample to patients who were admitted to hospitals that had continuously provided data to the database from 2011 to 2020. **Figure S10.** Non-linear associations between the FFP to RBC ratio or platelet to RBC ratio and adverse events in the sensitivity analyses with restricted cubic spline analysis conducted by restricting the sample to patients who were admitted to hospitals that had continuously provided data to the database from 2011 to 2020.

## Data Availability

Data used in the manuscript will not be made available because the datasets analyzed in the present study are not publicly available because of contracts with the hospitals providing data for the database.

## Abbreviations

RBCs: Red blood cells
FFP: Fresh frozen plasma
ICD-10: International Classification of Diseases, 10th Revision
