# Supplementary material for: Trends in massive transfusion practice for trauma in Japan from 2011 to 2020: a nationwide inpatient database study
Source: J Intensive Care. 2023 Oct 18;11:46. doi: 10.1186/s40560-023-00685-0 (PMC10585788; doi:10.1186/s40560-023-00685-0)
Supplement: Supplementary file 1 — Additional file 1: Table S1. ICD-10 codes for injured regions. Table S2. ICD-10 codes for adverse events. Table S3. Results for each of the complications. Table S4. Trends in the characteristics and outcomes of trauma patients requiring massive transfusion. Table S5. Trends in the incidence, blood products transfused, and outcomes of the sensitivity analysis by excluding 157 patients who died in the emergency room (N = 5090). Table S6. Results of the sensitivity analysis with generalized estimating equations to assess the association between the four transfusion ratio categories and outcomes conducted by excluding 157 patients who died in the emergency room (N = 5090). Table S7. Trends in the incidence, blood products transfused, and outcomes of the post hoc sensitivity analysis by altering the definition of massive transfusion to patients who received at least 20 units of RBC on the day of admission (N = 3238). Table S8. Results of the sensitivity analysis with generalized estimating equations to assess the association between the four transfusion ratio categories and outcomes conducted by altering the definition of massive transfusion to patients who received at least 20 units of RBC on the day of admission (N = 3238). Table S9. Trends in the incidence, blood products transfused, and outcomes of the post hoc sensitivity analysis by altering the definition of massive transfusion to patients who received at least 60 total units of RBC, FFP, and platelets within the first 2 days of admission (N = 4624). Table S10. Results of the sensitivity analysis with generalized estimating equations to assess the association between the four transfusion ratio categories and outcomes conducted by altering the definition of massive transfusion to patients who received at least 60 total units of RBC, FFP, and platelets within the first 2 days of admission (N = 4624). Table S11. Trends in the incidence, blood products transfused, and outcomes of the post hoc sensitivity analysis by r [file 40560_2023_685_MOESM1_ESM.docx]

**Additional file**

**Trends in massive transfusion practice for trauma in Japan from 2011–2020: A nationwide inpatient database study**

Hiroyuki Ohbe^1,2^, Takashi Tagami^1,3^, Akira Endo^4^, Shigeki Miyata^5^, Hiroki Matsui^1^, Kiyohide Fushimi^6^, Shigeki Kushimoto^2^, Hideo Yasunaga^1^

^1^Department of Clinical Epidemiology and Health Economics, School of Public Health, The University of Tokyo, Tokyo, Japan

^2^Division of Emergency and Critical Care Medicine, Tohoku University Graduate School of Medicine, Sendai, Japan

^3^Department of Emergency and Critical Care Medicine, Nippon Medical School Musashikosugi Hospital, Kanagawa, Japan

^4^Department of Acute Critical Care Medicine, Tsuchiura Kyodo General Hospital, Tsuchiura, Japan

^5^Central Blood Institute, Blood Service Headquarters, Japanese Red Cross Society, Tokyo, Japan

^6^Department of Health Policy and Informatics, Tokyo Medical and Dental University Graduate School, Tokyo, Japan

**List of Supplemental Tables and Figures**

**Supplemental Tables:**

**Table S1** ICD-10 codes for injured regions

**Table S2** ICD-10 codes for adverse events

**Table S3** Results for each of the complications

**Table S4** Trends in the characteristics and outcomes of trauma patients requiring massive transfusion

**Table S5** Trends in the incidence, blood products transfused, and outcomes of the sensitivity analysis by excluding 157 patients who died in the emergency room (N=5,090)

**Table S6** Results of the sensitivity analysis with generalized estimating equations to assess the association between the four transfusion ratio categories and outcomes conducted by excluding 157 patients who died in the emergency room (N=5,090)

**Table S7** Trends in the incidence, blood products transfused, and outcomes of the post-hoc sensitivity analysis by altering the definition of massive transfusion to patients who received at least 20 units of RBC on the day of admission (N=3,238)

**Table S8** Results of the sensitivity analysis with generalized estimating equations to assess the association between the four transfusion ratio categories and outcomes conducted by altering the definition of massive transfusion to patients who received at least 20 units of RBC on the day of admission (N=3,238)

**Table S9** Trends in the incidence, blood products transfused, and outcomes of the post-hoc sensitivity analysis by altering the definition of massive transfusion to patients who received at least 60 total units of RBC, FFP, and platelets within the first 2 days of admission (N=4,624)

**Table S10** Results of the sensitivity analysis with generalized estimating equations to assess the association between the four transfusion ratio categories and outcomes conducted by altering the definition of massive transfusion to patients who received at least 60 total units of RBC, FFP, and platelets within the first 2 days of admission (N=4,624)

**Table S11** Trends in the incidence, blood products transfused, and outcomes of the post-hoc sensitivity analysis by restricting the sample to patients admitted to the tertiary emergency centers (N=4,650)

**Table S12** Results of the sensitivity analysis with generalized estimating equations to assess the association between the four transfusion ratio categories and outcomes conducted by restricting the sample to patients admitted to the tertiary emergency centers (N=4,650)

**Table S13** Trends in the incidence, blood products transfused, and outcomes of the post-hoc sensitivity analysis by restricting the sample to patients who were admitted to hospitals that had continuously provided data to the database from 2011 to 2020 (N=3,783)

**Table S14** Results of the sensitivity analysis with generalized estimating equations to assess the association between the four transfusion ratio categories and outcomes conducted by restricting the sample to patients who were admitted to hospitals that had continuously provided data to the database from 2011 to 2020 (N=3,783)

**Supplemental Figures:**

**Figure S1** Non-linear associations between the FFP to RBC ratio or platelet to RBC ratio and in-hospital mortality revealed by sensitivity analyses with restricted cubic spline analysis conducted by excluding 157 patients who died in the emergency room

**Figure S2** Non-linear associations between the FFP to RBC ratio or platelet to RBC ratio and adverse events in the sensitivity analyses with restricted cubic spline analysis conducted by excluding 157 patients who died in the emergency room

**Figure S3** Non-linear associations between the FFP to RBC ratio or platelet to RBC ratio and in-hospital mortality revealed by sensitivity analyses with restricted cubic spline analysis conducted by altering the definition of massive transfusion to patients who received at least 20 units of RBC on the day of admission

**Figure S4** Non-linear associations between the FFP to RBC ratio or platelet to RBC ratio and adverse events in the sensitivity analyses with restricted cubic spline analysis conducted by altering the definition of massive transfusion to patients who received at least 20 units of RBC on the day of admission

**Figure S5** Non-linear associations between the FFP to RBC ratio or platelet to RBC ratio and in-hospital mortality revealed by sensitivity analyses with restricted cubic spline analysis conducted by altering the definition of massive transfusion to patients who received at least 60 total units of RBC, FFP, and platelets within the first 2 days of admission

**Figure S6** Non-linear associations between the FFP to RBC ratio or platelet to RBC ratio and adverse events in the sensitivity analyses with restricted cubic spline analysis conducted by altering the definition of massive transfusion to patients who received at least 60 total units of RBC, FFP, and platelets within the first 2 days of admission

**Figure S7** Non-linear associations between the FFP to RBC ratio or platelet to RBC ratio and in-hospital mortality revealed by sensitivity analyses with restricted cubic spline analysis conducted by restricting the sample to patients admitted to the tertiary emergency centers

**Figure S8** Non-linear associations between the FFP to RBC ratio or platelet to RBC ratio and adverse events in the sensitivity analyses with restricted cubic spline analysis conducted by restricting the restricting the sample to patients admitted to the tertiary emergency centers

**Figure S9** Non-linear associations between the FFP to RBC ratio or platelet to RBC ratio and in-hospital mortality revealed by sensitivity analyses with restricted cubic spline analysis conducted by restricting the sample to patients who were admitted to hospitals that had continuously provided data to the database from 2011 to 2020

**Figure S10** Non-linear associations between the FFP to RBC ratio or platelet to RBC ratio and adverse events in the sensitivity analyses with restricted cubic spline analysis conducted by restricting the sample to patients who were admitted to hospitals that had continuously provided data to the database from 2011 to 2020

**Table S1** ICD-10 codes for injured regions

| Injured resion, n (%) | ICD-10 codes |
| --- | --- |
| Head | S00–S09 |
| Neck | S10–S19 |
| Thorax | S20–S29 |
| Abdomen and pelvis | S30–S39, T08, T09 |
| Extremities | S40–S99, T10–T13 |
| Multiple | T00–T07, T14 |

ICD-10, International Classification of Diseases, 10^th^ Revision

**Table S2** ICD-10 codes for adverse events

| Post-admission diagnosis | ICD-10 codes |
| --- | --- |
| Cardiac failure | I110, I130, I132, I50 |
| Respiratory failure | J80, J81, J96, J960, J969, J984, J988, J989, J99 |
| Hepatic failure | K720, K729, K767, K778 |
| Renal failure | N141, N142, N17 |
| Sepsis | A021, A207, A227, A267, A327, A40, A41, A427, B377 |
| Thrombosis | D735, I21, I22, I26, I63 |
| Transfusion transmitted viral infections | B15–B17, B19 |
| Allergic/anaphylactic reactions | L500, T782, T805, T806, T886, I74, I81, I82, K550, K558, K559, K763, K765, B230, Z205, Z206, Z21 |
| Hemolytic transfusion reaction | D594, D596, D598 |
| Volume overload (other than the above) | E877, J90, J91, Y630 |
| Others | T800–804, T808, T809, Y446, Y449, Y598, Y599 |

ICD-10, International Classification of Diseases, 10^th^ Revision

**Table S3** Results for each of the complications

|  | Overall |
| --- | --- |
|  | (n=5,247) |
| Overall adverse events, n (%) | 1,080 (20.6) |
| Cardiac failure | 81 (1.5) |
| Respiratory failure | 430 (8.2) |
| Hepatic failure | 43 (0.8) |
| Renal failure | 294 (5.6) |
| Sepsis | 185 (3.5) |
| Thrombosis | 104 (2.0) |
| Transfusion transmitted viral infections | 7 (0.1) |
| Allergic/anaphylactic reactions | 85 (1.6) |
| Hemolytic transfusion reaction | 3 (0.1) |
| Volume overload (other than the above) | 79 (1.5) |
| Others | 1 (0.0) |

**Table S4** Trends in the characteristics and outcomes of trauma patients requiring massive transfusion

|  | Calendar year | | | | | | | | | |  |
| --- | --- | --- | --- | --- | --- | --- | --- | --- | --- | --- | --- |
|  | 2011 | 2012 | 2013 | 2014 | 2015 | 2016 | 2017 | 2018 | 2019 | 2020 | P for |
|  | N=532 | N=524 | N=585 | N=594 | N=571 | N=586 | N=517 | N=501 | N=473 | N=364 | trend |
| Hospital characteristics |  |  |  |  |  |  |  |  |  |  |  |
| Tertiary emergency hospital, % | 87.0 | 87.4 | 89.1 | 90.1 | 87.4 | 88.9 | 90.1 | 88.0 | 89.0 | 89.3 | 0.29 |
| Teaching hospital, % | 99.6 | 99.8 | 99.8 | 99.8 | 100.0 | 99.8 | 100.0 | 99.8 | 99.8 | 99.5 | 0.87 |
| Age, years, mean | 55.0 | 57.3 | 57.1 | 56.4 | 56.1 | 55.8 | 58.6 | 57.4 | 58.9 | 57.0 | 0.25 |
| Male, % | 65.8 | 63.0 | 62.4 | 64.0 | 65.3 | 61.1 | 64.0 | 63.1 | 68.7 | 63.2 | 0.67 |
| Body mass index at admission, kg/m^2^, % |  |  |  |  |  |  |  |  |  |  |  |
| <18.5 | 10.7 | 11.3 | 9.9 | 10.4 | 9.6 | 11.1 | 7.9 | 9.4 | 8.2 | 8.2 | 0.037 |
| 18.5–24.9 | 51.1 | 50.4 | 47.4 | 47.3 | 51.8 | 50.2 | 51.3 | 50.7 | 46.9 | 48.6 | 0.72 |
| 25.0–29.9 | 10.7 | 12.8 | 16.4 | 15.2 | 15.4 | 15.9 | 16.1 | 14.2 | 16.3 | 17.9 | 0.008 |
| ≥30.0 | 2.4 | 2.5 | 2.1 | 5.4 | 3.3 | 2.7 | 3.3 | 3.4 | 5.3 | 3.3 | 0.058 |
| Missing | 25.0 | 23.1 | 24.3 | 21.7 | 19.8 | 20.1 | 21.5 | 22.4 | 23.3 | 22.0 | 0.24 |
| Japan Coma Scale at admission, % |  |  |  |  |  |  |  |  |  |  |  |
| Alert | 20.9 | 25.2 | 23.6 | 23.4 | 18.2 | 17.9 | 17.4 | 20.4 | 18.6 | 15.4 | <0.001 |
| Confusion | 19.4 | 17.2 | 19.5 | 18.2 | 20.5 | 19.5 | 18.8 | 19.0 | 19.5 | 21.2 | 0.41 |
| Somnolence | 16.5 | 16.6 | 13.7 | 15.8 | 15.9 | 15.5 | 15.1 | 16.0 | 15.6 | 15.1 | 0.75 |
| Coma | 43.2 | 41.0 | 43.2 | 42.6 | 45.4 | 47.1 | 48.7 | 44.7 | 46.3 | 48.4 | 0.007 |
| Charlson comorbidity index, mean | 0.2 | 0.1 | 0.2 | 0.2 | 0.2 | 0.2 | 0.2 | 0.2 | 0.2 | 0.2 | 0.13 |
| Ambulance use, % | 93.8 | 91.2 | 96.2 | 94.3 | 94.9 | 95.4 | 97.5 | 97.2 | 94.5 | 96.7 | <0.001 |
| Injured region, % |  |  |  |  |  |  |  |  |  |  |  |
| Head | 19.2 | 15.6 | 15.4 | 17.0 | 18.0 | 16.7 | 15.3 | 15.4 | 14.6 | 18.7 | 0.42 |
| Neck | 2.4 | 1.5 | 3.1 | 2.0 | 1.2 | 2.4 | 1.4 | 1.2 | 2.5 | 1.4 | 0.25 |
| Thorax | 15.6 | 19.1 | 16.2 | 17.3 | 17.2 | 17.9 | 19.7 | 19.0 | 19.2 | 19.8 | 0.056 |
| Abdomen and pelvis | 46.6 | 47.7 | 50.9 | 49.5 | 51.5 | 50.0 | 51.8 | 52.7 | 51.2 | 48.9 | 0.11 |
| Extremities | 10.0 | 9.5 | 9.2 | 14.1 | 9.3 | 9.7 | 11.8 | 13.8 | 14.4 | 12.9 | 0.004 |
| Multiple | 26.9 | 25.2 | 26.8 | 20.4 | 26.1 | 24.9 | 24.2 | 23.6 | 18.0 | 23.1 | 0.009 |
| ICISS, mean | 2.5 | 2.4 | 2.6 | 2.3 | 2.7 | 2.7 | 2.7 | 2.9 | 2.8 | 2.8 | 0.006 |
| Treatment within 2 days of admission, % |  |  |  |  |  |  |  |  |  |  |  |
| Intensive care unit admission | 74.2 | 76.7 | 74.5 | 78.1 | 74.6 | 75.4 | 77.8 | 79.2 | 78.2 | 76.4 | 0.11 |
| High-dependency care unit admission | 23.5 | 20.2 | 25.3 | 21.7 | 24.9 | 24.2 | 21.7 | 20.4 | 22.0 | 24.2 | 0.73 |
| Surgery with general anesthesia | 73.3 | 71.0 | 68.4 | 68.5 | 70.4 | 67.9 | 71.6 | 67.7 | 69.6 | 70.9 | 0.38 |
| Interventional radiology | 36.7 | 37.4 | 41.2 | 39.9 | 42.9 | 44.0 | 46.4 | 44.3 | 44.2 | 44.8 | <0.001 |
| Mechanical ventilation | 80.3 | 79.0 | 80.0 | 78.5 | 78.1 | 78.7 | 77.2 | 76.6 | 80.8 | 78.3 | 0.41 |
| Vasopressors | 83.6 | 86.8 | 87.0 | 83.7 | 86.9 | 88.4 | 91.1 | 89.8 | 93.9 | 92.6 | <0.001 |
| Outcomes |  |  |  |  |  |  |  |  |  |  |  |
| In-hospital mortality, % | 43.4 | 37.2 | 40.5 | 37.5 | 38.7 | 38.6 | 38.5 | 36.9 | 43.6 | 41.2 | 0.96 |
| Adverse events, % | 18.6 | 17.6 | 21.2 | 19.5 | 19.6 | 23.4 | 20.7 | 21.2 | 22.6 | 22.0 | 0.026 |
| Death in the emergency or operating room, % | 1.9 | 2.7 | 3.1 | 2.7 | 2.5 | 3.9 | 3.1 | 3.6 | 4.0 | 2.5 | 0.14 |
| Death within 24 h, % | 21.2 | 17.7 | 22.1 | 19.4 | 18.9 | 18.1 | 20.7 | 19.4 | 20.1 | 19 | 0.63 |
| Duration of hospitalization, days, mean | 51.5 | 51.5 | 47.3 | 46.9 | 51.1 | 48.1 | 53.1 | 49.4 | 46.8 | 40.2 | 0.089 |
| Hospitalization costs, thousand yen, mean | 5030 | 5266 | 5201 | 5502 | 5774 | 5828 | 6060 | 5980 | 5935 | 5912 | 0.006 |

SD, standard deviation; ICDISS, International Classification of Diseases, Tenth Revision–Based Injury Severity Score

**Table S5** Trends in the incidence, blood products transfused, and outcomes of the sensitivity analysis by excluding 157 patients who died in the emergency room (N=5,090)

|  | Calendar year | | | | | | | | | |  |
| --- | --- | --- | --- | --- | --- | --- | --- | --- | --- | --- | --- |
|  | 2011 | 2012 | 2013 | 2014 | 2015 | 2016 | 2017 | 2018 | 2019 | 2020 | P for |
| Number of trauma requiring MT, n | 522 | 510 | 567 | 578 | 557 | 563 | 501 | 483 | 454 | 355 | 0.025 |
| Incidence of MT, % | 0.23 | 0.16 | 0.17 | 0.16 | 0.15 | 0.14 | 0.12 | 0.12 | 0.12 | 0.10 | <0.001 |
| RBC, unit, mean | 32.3 | 31.4 | 32.0 | 30.5 | 31.6 | 31.9 | 32.9 | 31.8 | 33.0 | 31.6 | 0.65 |
| FFP, unit, mean | 26.6 | 26.9 | 27.8 | 27.4 | 29.7 | 31.9 | 33.2 | 33.1 | 36.0 | 34.8 | <0.001 |
| Platelets, unit, mean | 22.6 | 23.7 | 24.5 | 24.5 | 26.7 | 27.7 | 27.7 | 25.9 | 27.2 | 25.2 | 0.060 |
| FFP to RBC ratio, mean | 0.82 | 0.86 | 0.86 | 0.90 | 0.93 | 1.02 | 1.01 | 1.05 | 1.10 | 1.11 | <0.001 |
| Platelets to RBC ratio, mean | 0.73 | 0.77 | 0.76 | 0.80 | 0.85 | 0.88 | 0.85 | 0.83 | 0.83 | 0.81 | 0.089 |
| In-hospital mortality, % | 42.3 | 35.5 | 38.6 | 35.8 | 37.2 | 36.1 | 36.5 | 34.6 | 41.2 | 39.7 | 0.93 |
| Adverse events, % | 19.0 | 17.8 | 21.9 | 20.1 | 20.1 | 24.2 | 21.4 | 21.9 | 23.3 | 22.3 | 0.016 |

MT, massive transfusion; RBC, red blood cell; FFP, fresh frozen plasma

**Table S6** Results of the sensitivity analysis with generalized estimating equations to assess the association between the four transfusion ratio categories and outcomes conducted by excluding 157 patients who died in the emergency room (N=5,090)

|  | In-hospital mortality | | |  | Adverse events | | |
| --- | --- | --- | --- | --- | --- | --- | --- |
|  | Number  (%) | Adjusted odds ratio  (95% CI) | P  value |  | Number  (%) | Adjusted odds ratio  (95% CI) | P  value |
| FFP to RBC ratio |  |  |  |  |  |  |  |
| –0.75 | 629/1546 (40.7) | 1.22 (1.03–1.44) | 0.019 |  | 257/1546 (16.6) | 0.81 (0.67–0.97) | 0.021 |
| 0.75–1.00 | 605/1690 (35.8) | Refefence | – |  | 346/1690 (20.5) | Refefence | – |
| 1.00–1.25 | 324/943 (34.4) | 0.97 (0.80–1.17) | 0.77 |  | 225/943 (23.9) | 1.20 (0.99–1.46) | 0.063 |
| 1.25– | 358/911 (39.3) | 1.12 (0.93–1.36) | 0.24 |  | 248/911 (27.2) | 1.47 (1.21–1.78) | <0.001 |
| Platelet to RBC ratio |  |  |  |  |  |  |  |
| –0.75 | 1025/2271 (45.1) | 1.84 (1.58–2.15) | <0.001 |  | 372/2271 (16.4) | 0.64 (0.54–0.75) | <0.001 |
| 0.75–1.00 | 505/1562 (32.3) | Refefence | – |  | 373/1562 (23.9) | Refefence | – |
| 1.00–1.25 | 163/493 (33.1) | 1.17 (0.92–1.48) | 0.21 |  | 127/493 (25.8) | 1.11 (0.88–1.41) | 0.38 |
| 1.25– | 223/764 (29.2) | 0.86 (0.69–1.06) | 0.15 |  | 204/764 (26.7) | 1.16 (0.95–1.42) | 0.15 |

The model was adjusted for calendar year at admission, hospital characteristics; age, sex, and body mass index at admission; Japan Coma Scale at admission; Charlson comorbidity index; ambulance use; injured regions; and ICD-10–based injury severity score as covariates.

CI, confidence interval; RBC, red blood cell; FFP, fresh frozen plasma; ICD-10, International Classification of Diseases, 10^th^ Revision

**Table S7** Trends in the incidence, blood products transfused, and outcomes of the post-hoc sensitivity analysis by altering the definition of massive transfusion to patients who received at least 20 units of RBC on the day of admission (N=3,238)

|  | Calendar year | | | | | | | | | |  |
| --- | --- | --- | --- | --- | --- | --- | --- | --- | --- | --- | --- |
|  | 2011 | 2012 | 2013 | 2014 | 2015 | 2016 | 2017 | 2018 | 2019 | 2020 | P for |
| Number of trauma requiring MT, n | 342 | 328 | 345 | 360 | 371 | 356 | 318 | 312 | 298 | 208 | 0.060 |
| Incidence of MT, % | 0.15 | 0.11 | 0.11 | 0.10 | 0.10 | 0.09 | 0.08 | 0.08 | 0.08 | 0.06 | <0.001 |
| RBC, unit, mean | 30.8 | 30.3 | 31.7 | 29.8 | 30.5 | 29.6 | 31.6 | 30.5 | 30.9 | 32.1 | 0.42 |
| FFP, unit, mean | 23.6 | 24.1 | 25.7 | 25.2 | 26.8 | 27.4 | 30.3 | 29.9 | 31.5 | 33.3 | <0.001 |
| Platelets, unit, mean | 17.9 | 18.8 | 20.1 | 20.7 | 21.4 | 20.3 | 22.8 | 20.4 | 21.3 | 21.1 | 0.025 |
| FFP to RBC ratio, mean | 0.76 | 0.80 | 0.81 | 0.85 | 0.88 | 0.94 | 0.96 | 1.00 | 1.03 | 1.05 | <0.001 |
| Platelets to RBC ratio, mean | 0.61 | 0.63 | 0.64 | 0.69 | 0.70 | 0.69 | 0.74 | 0.68 | 0.69 | 0.67 | 0.25 |
| In-hospital mortality, % | 50.9 | 42.7 | 49.6 | 44.7 | 45.3 | 46.9 | 48.4 | 42.9 | 47.7 | 48.1 | 0.93 |
| Adverse events, % | 18.7 | 15.9 | 18.6 | 16.1 | 16.7 | 19.1 | 20.1 | 18.9 | 18.1 | 20.2 | 0.089 |

MT, massive transfusion; RBC, red blood cell; FFP, fresh frozen plasma

**Table S8** Results of the sensitivity analysis with generalized estimating equations to assess the association between the four transfusion ratio categories and outcomes conducted by altering the definition of massive transfusion to patients who received at least 20 units of RBC on the day of admission (N=3,238)

|  | In-hospital mortality | | |  | Adverse events | | |
| --- | --- | --- | --- | --- | --- | --- | --- |
|  | Number  (%) | Adjusted odds ratio  (95% CI) | P  value |  | Number  (%) | Adjusted odds ratio  (95% CI) | P  value |
| FFP to RBC ratio |  |  |  |  |  |  |  |
| –0.75 | 607/1144 (53.1) | 1.29 (1.06–1.57) | 0.012 |  | 166/1144 (14.5) | 0.78 (0.62–0.97) | 0.028 |
| 0.75–1.00 | 500/1120 (44.6) | Reference | – |  | 214/1120 (19.1) | Reference | – |
| 1.00–1.25 | 210/492 (42.7) | 0.87 (0.67–1.11) | 0.26 |  | 106/492 (21.5) | 1.19 (0.91–1.55) | 0.20 |
| 1.25– | 194/482 (40.3) | 0.78 (0.60–1.01) | 0.060 |  | 101/482 (21.0) | 1.12 (0.85–1.48) | 0.40 |
| Platelet to RBC ratio |  |  |  |  |  |  |  |
| –0.75 | 1012/1810 (55.9) | 2.36 (1.94–2.87) | <0.001 |  | 270/1810 (14.9) | 0.73 (0.59–0.90) | 0.003 |
| 0.75–1.00 | 326/927 (35.2) | Reference | – |  | 188/927 (20.3) | Reference | – |
| 1.00–1.25 | 79/222 (35.6) | 1.11 (0.78–1.58) | 0.56 |  | 62/222 (27.9) | 1.58 (1.13–2.23) | 0.008 |
| 1.25– | 94/279 (33.7) | 0.96 (0.70–1.32) | 0.80 |  | 67/279 (24.0) | 1.26 (0.91–1.73) | 0.17 |

The model was adjusted for calendar year at admission, hospital characteristics; age, sex, and body mass index at admission; Japan Coma Scale at admission; Charlson comorbidity index; ambulance use; injured regions; and ICD-10–based injury severity score as covariates.

CI, confidence interval; RBC, red blood cell; FFP, fresh frozen plasma; ICD-10, International Classification of Diseases, 10^th^ Revision

**Table S9** Trends in the incidence, blood products transfused, and outcomes of the post-hoc sensitivity analysis by altering the definition of massive transfusion to patients who received at least 60 total units of RBC, FFP, and platelets within the first 2 days of admission (N=4,624)

|  | Calendar year | | | | | | | | | |  |
| --- | --- | --- | --- | --- | --- | --- | --- | --- | --- | --- | --- |
|  | 2011 | 2012 | 2013 | 2014 | 2015 | 2016 | 2017 | 2018 | 2019 | 2020 | P for |
| Number of trauma requiring MT, n | 399 | 419 | 472 | 488 | 498 | 544 | 493 | 473 | 464 | 387 | 0.79 |
| Incidence of MT, % | 0.18 | 0.14 | 0.14 | 0.13 | 0.13 | 0.13 | 0.12 | 0.12 | 0.13 | 0.11 | <0.001 |
| RBC, unit, mean | 34.2 | 32.0 | 32.9 | 31.0 | 31.6 | 31.3 | 31.5 | 31.0 | 31.3 | 29.2 | 0.006 |
| FFP, unit, mean | 31.8 | 31.6 | 31.8 | 31.0 | 32.9 | 34.0 | 35.3 | 35.2 | 37.1 | 35.4 | 0.003 |
| Platelets, unit, mean | 30.7 | 30.1 | 31.5 | 31.5 | 33.9 | 32.1 | 33.1 | 30.9 | 32.0 | 30.0 | 0.79 |
| FFP to RBC ratio, mean | 1.01 | 1.10 | 1.05 | 1.08 | 1.10 | 1.17 | 1.22 | 1.23 | 1.27 | 1.32 | <0.001 |
| Platelets to RBC ratio, mean | 1.12 | 1.15 | 1.18 | 1.37 | 1.26 | 1.20 | 1.30 | 1.24 | 1.24 | 1.43 | 0.040 |
| In-hospital mortality, % | 40.4 | 32.9 | 35.6 | 37.1 | 36.5 | 34.6 | 33.3 | 35.5 | 39.4 | 37.7 | 0.79 |
| Adverse events, % | 23.1 | 21.0 | 25.0 | 23.0 | 21.5 | 23.2 | 23.5 | 23.9 | 25.6 | 23.5 | 0.089 |

MT, massive transfusion; RBC, red blood cell; FFP, fresh frozen plasma

**Table S10** Results of the sensitivity analysis with generalized estimating equations to assess the association between the four transfusion ratio categories and outcomes conducted by altering the definition of massive transfusion to patients who received at least 60 total units of RBC, FFP, and platelets within the first 2 days of admission (N=4,624)

|  | In-hospital mortality | | |  | Adverse events | | |
| --- | --- | --- | --- | --- | --- | --- | --- |
|  | Number  (%) | Adjusted odds ratio  (95% CI) | P  value |  | Number  (%) | Adjusted odds ratio  (95% CI) | P  value |
| FFP to RBC ratio |  |  |  |  |  |  |  |
| –0.75 | 343/816 (42.0) | 1.26 (1.03–1.54) | 0.022 |  | 159/816 (19.5) | 0.86 (0.70–1.06) | 0.17 |
| 0.75–1.00 | 523/1441 (36.3) | Reference | – |  | 315/1441 (21.9) | Reference | – |
| 1.00–1.25 | 328/969 (33.9) | 0.93 (0.77–1.12) | 0.45 |  | 227/969 (23.4) | 1.10 (0.90–1.33) | 0.36 |
| 1.25– | 483/1398 (34.6) | 0.91 (0.76–1.09) | 0.29 |  | 374/1398 (26.8) | 1.34 (1.12–1.59) | 0.001 |
| Platelet to RBC ratio |  |  |  |  |  |  |  |
| –0.75 | 676/1316 (51.4) | 2.11 (1.76–2.53) | <0.001 |  | 238/1316 (18.1) | 0.65 (0.54–0.78) | <0.001 |
| 0.75–1.00 | 429/1282 (33.5) | Reference | – |  | 318/1282 (24.8) | Reference | – |
| 1.00–1.25 | 207/676 (30.6) | 0.92 (0.74–1.14) | 0.45 |  | 163/676 (24.1) | 0.96 (0.78–1.19) | 0.72 |
| 1.25– | 365/1350 (27.0) | 0.66 (0.55–0.79) | <0.001 |  | 356/1350 (26.4) | 1.10 (0.92–1.31) | 0.29 |

The model was adjusted for calendar year at admission, hospital characteristics; age, sex, and body mass index at admission; Japan Coma Scale at admission; Charlson comorbidity index; ambulance use; injured regions; and ICD-10–based injury severity score as covariates.

CI, confidence interval; RBC, red blood cell; FFP, fresh frozen plasma; ICD-10, International Classification of Diseases, 10^th^ Revision

**Table S11** Trends in the incidence, blood products transfused, and outcomes of the post-hoc sensitivity analysis by restricting the sample to patients admitted to tertiary emergency centers (N=4,650)

|  | Calendar year | | | | | | | | | |  |
| --- | --- | --- | --- | --- | --- | --- | --- | --- | --- | --- | --- |
|  | 2011 | 2012 | 2013 | 2014 | 2015 | 2016 | 2017 | 2018 | 2019 | 2020 | P for |
| Number of trauma requiring MT, n | 463 | 458 | 521 | 535 | 499 | 521 | 466 | 441 | 421 | 325 | 0.073 |
| Incidence of MT, % | 0.20 | 0.15 | 0.16 | 0.15 | 0.13 | 0.13 | 0.12 | 0.11 | 0.11 | 0.09 | <0.001 |
| RBC, unit, mean | 32.4 | 31.5 | 32.3 | 30.8 | 31.8 | 31.8 | 32.8 | 32.4 | 32.9 | 32.0 | 0.98 |
| FFP, unit, mean | 26.8 | 26.8 | 28.3 | 28.0 | 30.2 | 31.9 | 33.6 | 34.0 | 36.1 | 35.7 | 0.002 |
| Platelets, unit, mean | 22.5 | 22.8 | 24.2 | 24.3 | 26.7 | 27.0 | 27.2 | 26.0 | 26.0 | 24.8 | 0.040 |
| FFP to RBC ratio, mean | 0.83 | 0.86 | 0.87 | 0.91 | 0.94 | 1.02 | 1.02 | 1.06 | 1.10 | 1.13 | <0.001 |
| Platelets to RBC ratio, mean | 0.72 | 0.74 | 0.75 | 0.79 | 0.84 | 0.86 | 0.84 | 0.82 | 0.79 | 0.79 | 0.13 |
| In-hospital mortality, % | 42.3 | 36.2 | 40.7 | 38.3 | 37.7 | 38.2 | 37.1 | 37.4 | 43.0 | 42.8 | 0.79 |
| Adverse events, % | 17.3 | 17.2 | 20.5 | 18.7 | 18.6 | 23.6 | 21.0 | 22.2 | 22.1 | 21.5 | 0.040 |

MT, massive transfusion; RBC, red blood cell; FFP, fresh frozen plasma

**Table S12** Results of the sensitivity analysis with generalized estimating equations to assess the association between the four transfusion ratio categories and outcomes conducted by restricting the sample to patients admitted to tertiary emergency centers (N=4,650)

|  | In-hospital mortality | | |  | Adverse events | | |
| --- | --- | --- | --- | --- | --- | --- | --- |
|  | Number  (%) | Adjusted odds ratio  (95% CI) | P  value |  | Number  (%) | Adjusted odds ratio  (95% CI) | P  value |
| FFP to RBC ratio |  |  |  |  |  |  |  |
| –0.75 | 588/1357 (43.3) | 1.22 (1.02–1.46) | 0.029 |  | 204/1357 (15.0) | 0.79 (0.65–0.96) | 0.021 |
| 0.75–1.00 | 579/1551 (37.3) | Reference | – |  | 303/1551 (19.5) | Reference | – |
| 1.00–1.25 | 315/886 (35.6) | 0.94 (0.77–1.15) | 0.54 |  | 206/886 (23.3) | 1.21 (0.99–1.49) | 0.061 |
| 1.25– | 342/856 (40.0) | 1.08 (0.88–1.32) | 0.46 |  | 228/856 (26.6) | 1.47 (1.20–1.81) | <0.001 |
| Platelet to RBC ratio |  |  |  |  |  |  |  |
| –0.75 | 1033/2150 (48.1) | 1.93 (1.64–2.28) | <0.001 |  | 328/2150 (15.3) | 0.62 (0.52–0.74) | <0.001 |
| 0.75–1.00 | 448/1382 (32.4) | Reference | – |  | 321/1382 (23.2) | Reference | – |
| 1.00–1.25 | 146/442 (33.0) | 1.16 (0.90–1.50) | 0.25 |  | 116/442 (26.2) | 1.18 (0.92–1.52) | 0.18 |
| 1.25– | 197/676 (29.1) | 0.85 (0.67–1.06) | 0.15 |  | 176/676 (26.0) | 1.18 (0.95–1.47) | 0.13 |

The model was adjusted for calendar year at admission, hospital characteristics; age, sex, and body mass index at admission; Japan Coma Scale at admission; Charlson comorbidity index; ambulance use; injured regions; and ICD-10–based injury severity score as covariates.

CI, confidence interval; RBC, red blood cell; FFP, fresh frozen plasma; ICD-10, International Classification of Diseases, 10^th^ Revision

**Table S13** Trends in the incidence, blood products transfused, and outcomes of the post-hoc sensitivity analysis by restricting the sample to patients who were admitted to hospitals that had continuously provided data to the database from 2011 to 2020 (N=3,783)

|  | Calendar year | | | | | | | | | |  |
| --- | --- | --- | --- | --- | --- | --- | --- | --- | --- | --- | --- |
|  | 2011 | 2012 | 2013 | 2014 | 2015 | 2016 | 2017 | 2018 | 2019 | 2020 | P for |
| Number of trauma requiring MT, n | 387 | 352 | 427 | 419 | 407 | 437 | 362 | 371 | 365 | 256 | 0.25 |
| Incidence of MT, % | 0.17 | 0.11 | 0.13 | 0.12 | 0.11 | 0.11 | 0.09 | 0.09 | 0.10 | 0.07 | <0.001 |
| RBC, unit, mean | 32.1 | 30.3 | 31.5 | 30.2 | 32.0 | 32.0 | 31.7 | 32.2 | 32.8 | 32.3 | 0.060 |
| FFP, unit, mean | 26.5 | 25.3 | 26.8 | 27.3 | 29.7 | 31.7 | 31.0 | 33.6 | 34.6 | 35.1 | <0.001 |
| Platelets, unit, mean | 22.9 | 22.7 | 23.7 | 24.0 | 26.2 | 26.9 | 24.8 | 26.1 | 25.8 | 24.8 | 0.060 |
| FFP to RBC ratio, mean | 0.82 | 0.85 | 0.85 | 0.91 | 0.92 | 1.00 | 0.97 | 1.05 | 1.06 | 1.10 | <0.001 |
| Platelets to RBC ratio, mean | 0.74 | 0.76 | 0.75 | 0.80 | 0.82 | 0.85 | 0.78 | 0.82 | 0.78 | 0.79 | 0.18 |
| In-hospital mortality, % | 41.6 | 35.8 | 40.5 | 38.4 | 37.8 | 36.4 | 36.5 | 34.2 | 41.9 | 41.0 | 0.79 |
| Adverse events, % | 19.4 | 19.3 | 21.1 | 19.6 | 20.1 | 23.8 | 20.4 | 21.6 | 21.4 | 19.5 | 0.18 |

MT, massive transfusion; RBC, red blood cell; FFP, fresh frozen plasma

**Table S14** Results of the sensitivity analysis with generalized estimating equations to assess the association between the four transfusion ratio categories and outcomes conducted by restricting the sample to patients who were admitted to hospitals that had continuously provided data to the database from 2011 to 2020 (N=3,783)

|  | In-hospital mortality | | |  | Adverse events | | |
| --- | --- | --- | --- | --- | --- | --- | --- |
|  | Number  (%) | Adjusted odds ratio  (95% CI) | P  value |  | Number  (%) | Adjusted odds ratio  (95% CI) | P  value |
| FFP to RBC ratio |  |  |  |  |  |  |  |
| –0.75 | 517/1195 (43.3) | 1.25 (1.03–1.52) | 0.027 |  | 183/1195 (15.3) | 0.72 (0.58–0.89) | 0.003 |
| 0.75–1.00 | 449/1239 (36.2) | Reference | – |  | 254/1239 (20.5) | Reference | – |
| 1.00–1.25 | 227/696 (32.6) | 0.90 (0.71–1.12) | 0.34 |  | 172/696 (24.7) | 1.25 (1.00–1.57) | 0.052 |
| 1.25– | 258/653 (39.5) | 1.13 (0.90–1.43) | 0.29 |  | 174/653 (26.7) | 1.43 (1.13–1.8) | 0.002 |
| Platelet to RBC ratio |  |  |  |  |  |  |  |
| –0.75 | 820/1753 (46.8) | 1.87 (1.55–2.25) | <0.001 |  | 273/1753 (15.6) | 0.59 (0.49–0.71) | <0.001 |
| 0.75–1.00 | 363/1131 (32.1) | Reference | – |  | 279/1131 (24.7) | Reference | – |
| 1.00–1.25 | 118/360 (32.8) | 1.22 (0.92–1.62) | 0.18 |  | 89/360 (24.7) | 1.01 (0.76–1.34) | 0.94 |
| 1.25– | 150/539 (27.8) | 0.78 (0.60–1.01) | 0.056 |  | 142/539 (26.4) | 1.12 (0.88–1.42) | 0.36 |

The model was adjusted for calendar year at admission, hospital characteristics; age, sex, and body mass index at admission; Japan Coma Scale at admission; Charlson comorbidity index; ambulance use; injured regions; and ICD-10–based injury severity score as covariates.

CI, confidence interval; RBC, red blood cell; FFP, fresh frozen plasma; ICD-10, International Classification of Diseases, 10^th^ Revision

**Figure S1** Non-linear associations between the FFP to RBC ratio or platelet to RBC ratio and in-hospital mortality revealed by sensitivity analyses with restricted cubic spline analysis conducted by excluding 157 patients who died in the emergency room


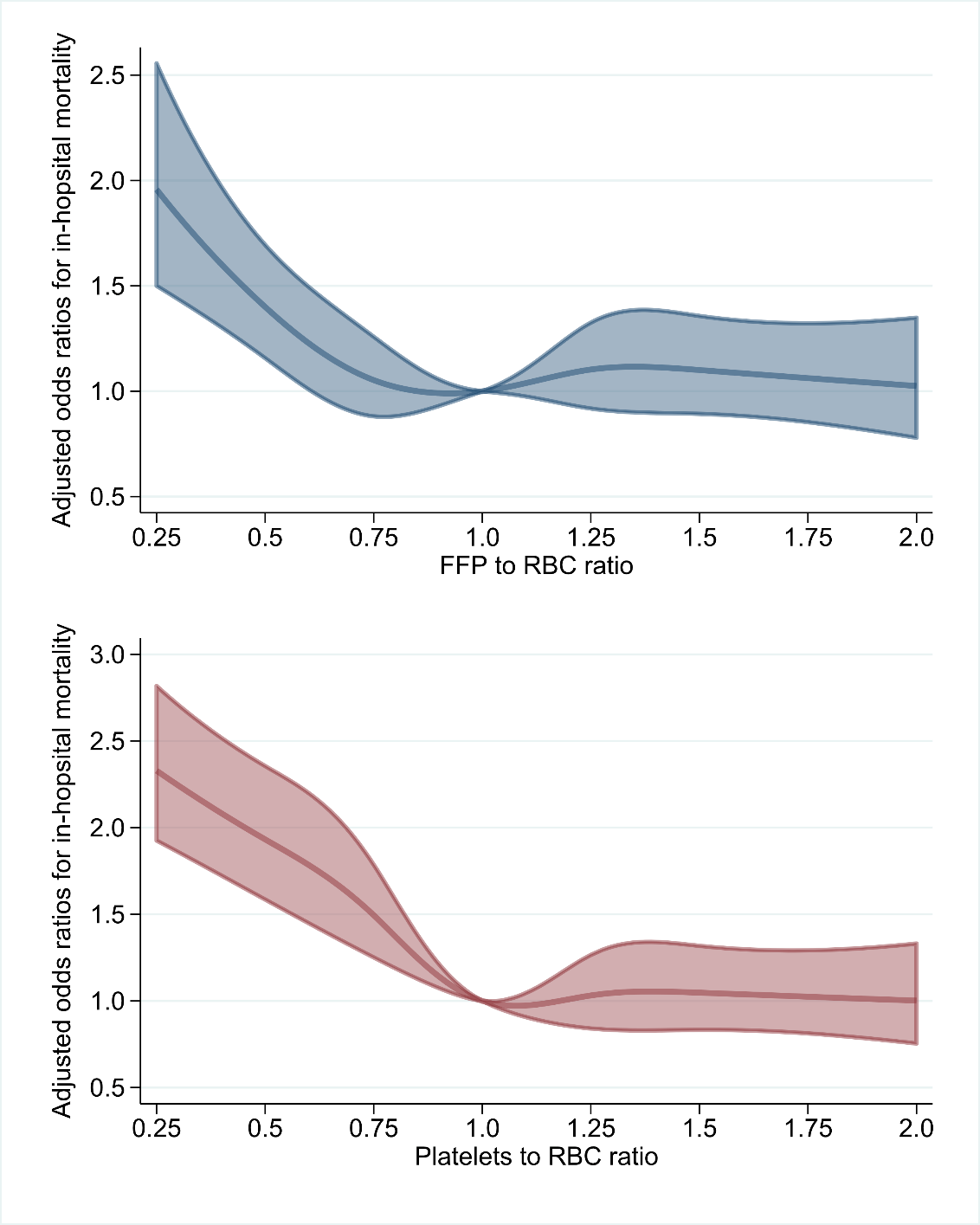


The model was adjusted for calendar year at admission; hospital characteristics; age, sex, and body mass index at admission; Japan Coma Scale at admission; Charlson comorbidity index; ambulance use; injured regions; and ICD-10–based injury severity score as covariates. Five transfusion ratio points (0.50, 0.75, 1.00, 1.25, and 1.50) were denoted as the knots and 1.00 was designated as the reference point. The area region represents 95% confidence intervals for the estimated adjusted odds ratios.

FFP, fresh frozen plasma; RBC, red blood cell; ICD-10, International Classification of Diseases, 10^th^ Revision

**Figure S2** Non-linear associations between the FFP to RBC ratio or platelet to RBC ratio and adverse events in the sensitivity analyses with restricted cubic spline analysis conducted by excluding 157 patients who died in the emergency room


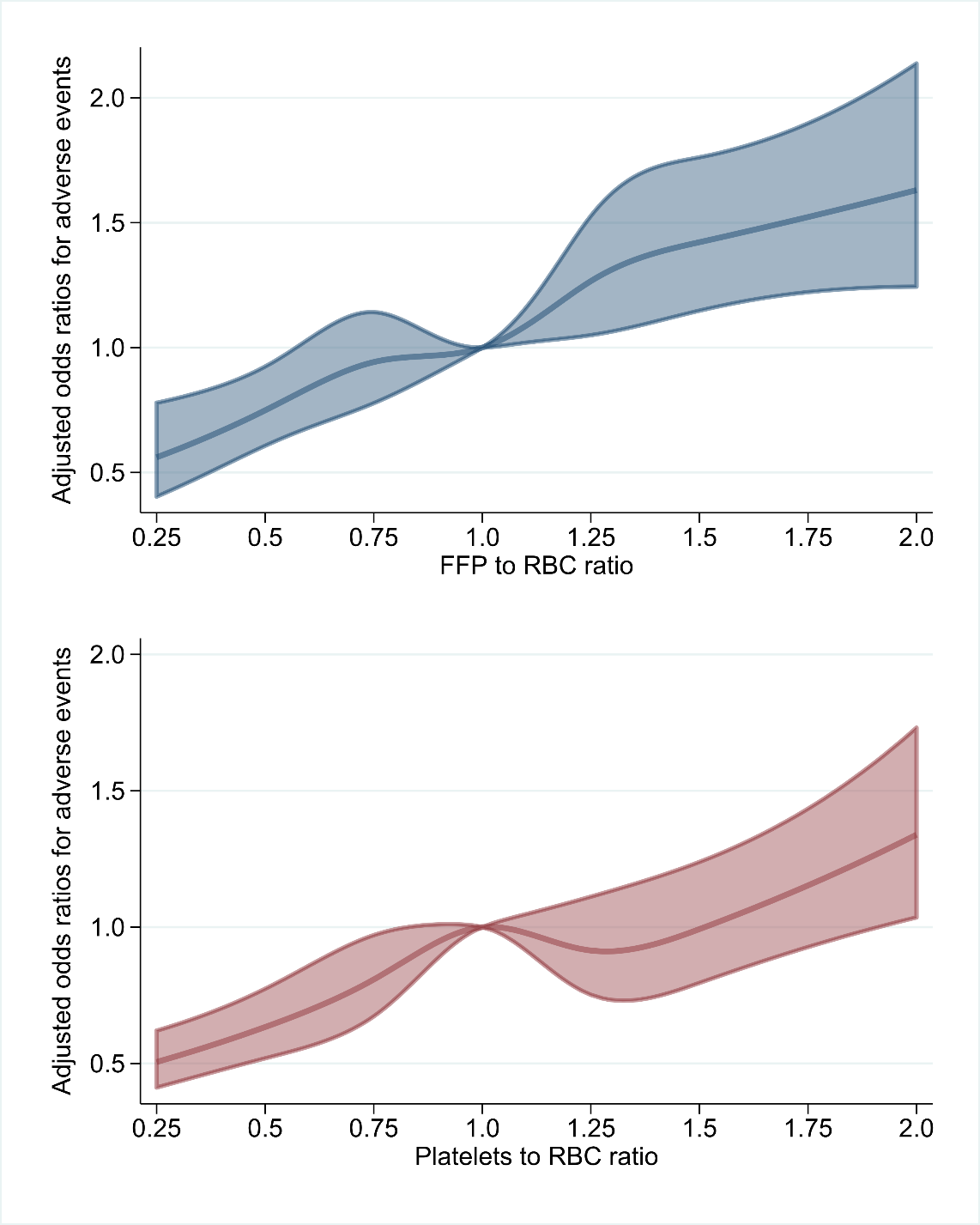


The model was adjusted for calendar year at admission; hospital characteristics; age, sex, and body mass index at admission; Japan Coma Scale at admission; Charlson comorbidity index score; ambulance use; injured regions; and ICD-10–based injury severity score as covariates. Five transfusion ratio points (0.50, 0.75, 1.00, 1.25, and 1.50) were denoted as the knots and 1.00 was designated as the reference category. The area region represents 95% confidence intervals for the estimated adjusted odds ratios.

FFP, fresh frozen plasma; RBC, red blood cell; ICD-10, International Classification of Diseases, 10^th^ Revision

**Figure S3** Non-linear associations between the FFP to RBC ratio or platelet to RBC ratio and in-hospital mortality revealed by sensitivity analyses with restricted cubic spline analysis conducted by altering the definition of massive transfusion to patients who received at least 20 units of RBC on the day of admission


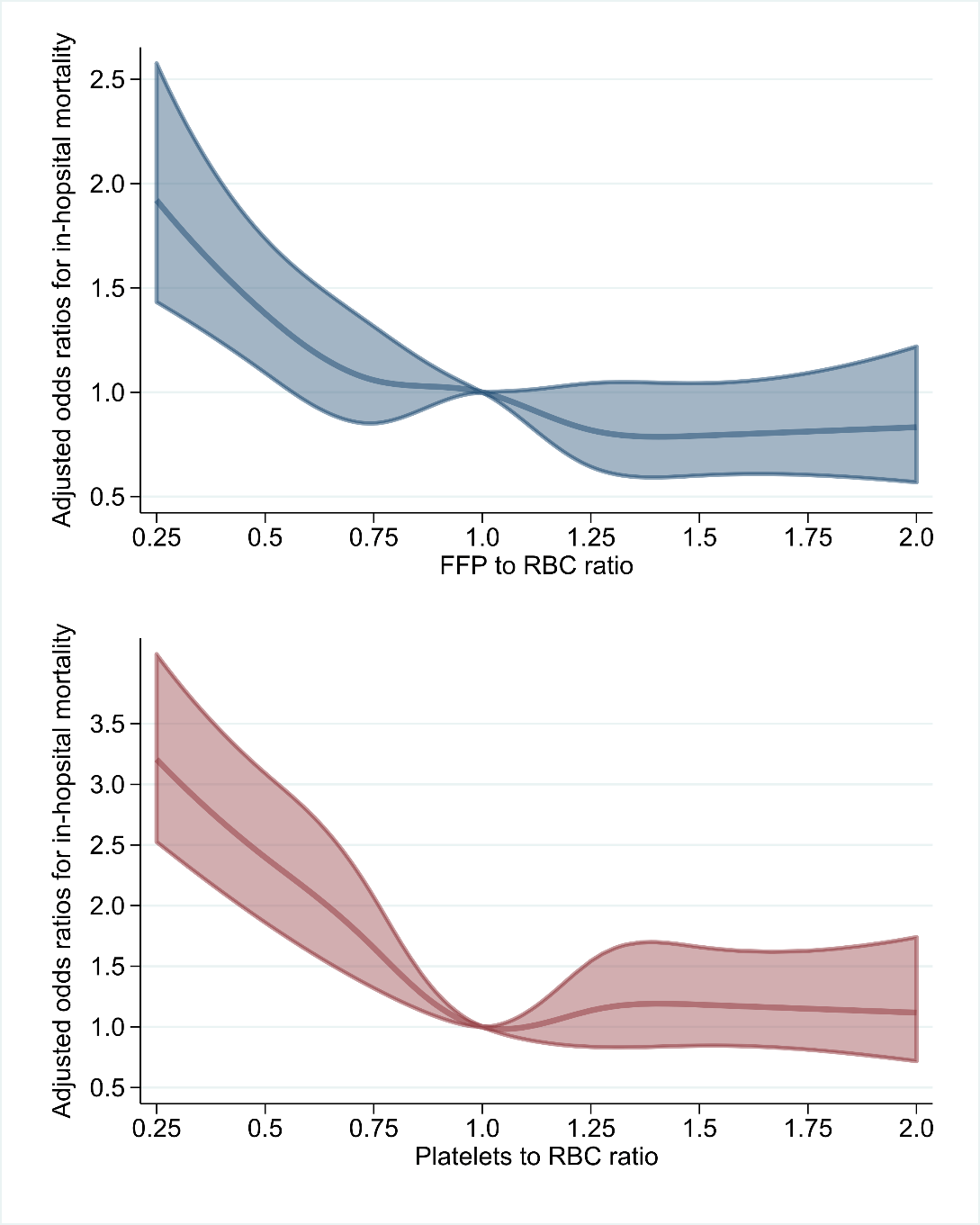


The model was adjusted for calendar year at admission; hospital characteristics; age, sex, and body mass index at admission; Japan Coma Scale at admission; Charlson comorbidity index; ambulance use; injured regions; and ICD-10–based injury severity score as covariates. Five transfusion ratio points (0.50, 0.75, 1.00, 1.25, and 1.50) were denoted as the knots and 1.00 was designated as the reference point. The area region represents 95% confidence intervals for the estimated adjusted odds ratios.

FFP, fresh frozen plasma; RBC, red blood cell; ICD-10, International Classification of Diseases, 10^th^ Revision

**Figure S4** Non-linear associations between the FFP to RBC ratio or platelet to RBC ratio and adverse events in the sensitivity analyses with restricted cubic spline analysis conducted by altering the definition of massive transfusion to patients who received at least 20 units of RBC on the day of admission


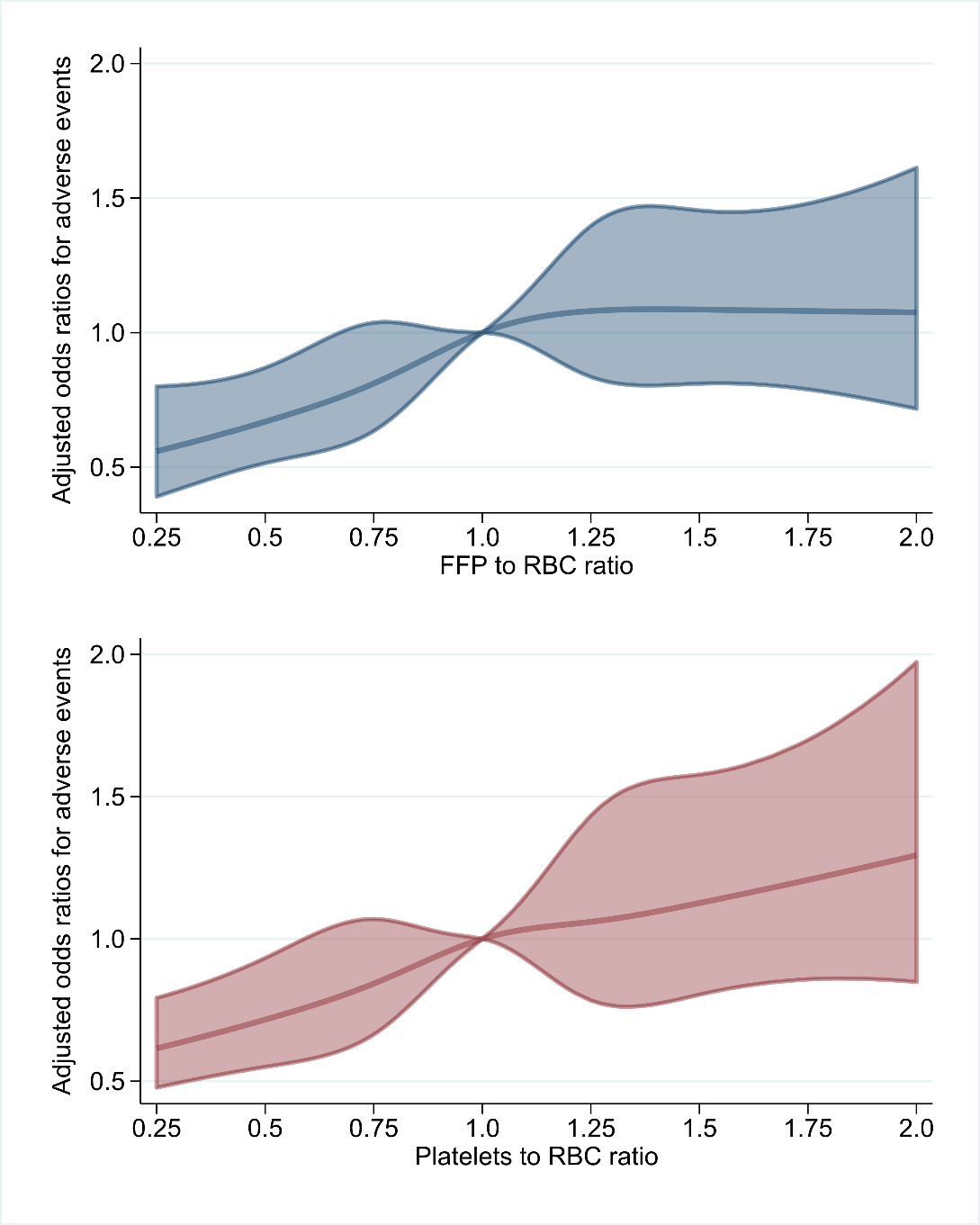


The model was adjusted for calendar year at admission; hospital characteristics; age, sex, and body mass index at admission; Japan Coma Scale at admission; Charlson comorbidity index score; ambulance use; injured regions; and ICD-10–based injury severity score as covariates. Five transfusion ratio points (0.50, 0.75, 1.00, 1.25, and 1.50) were denoted as the knots and 1.00 was designated as the reference category. The area region represents 95% confidence intervals for the estimated adjusted odds ratios.

FFP, fresh frozen plasma; RBC, red blood cell; ICD-10, International Classification of Diseases, 10^th^ Revision

**Figure S5** Non-linear associations between the FFP to RBC ratio or platelet to RBC ratio and in-hospital mortality revealed by sensitivity analyses with restricted cubic spline analysis conducted by altering the definition of massive transfusion to patients who received at least 60 total units of RBC, FFP, and platelets within the first 2 days of admission


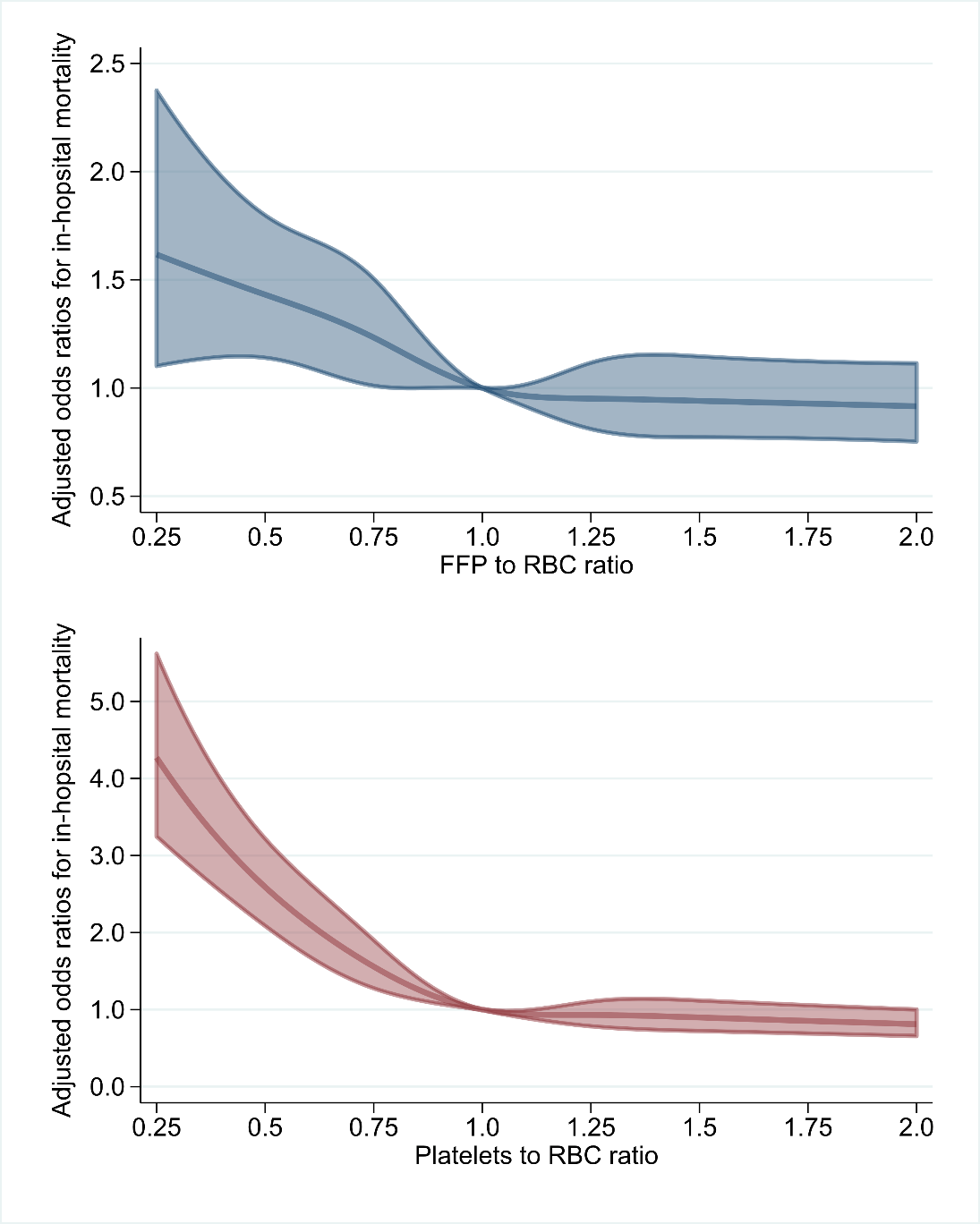


The model was adjusted for calendar year at admission; hospital characteristics; age, sex, and body mass index at admission; Japan Coma Scale at admission; Charlson comorbidity index; ambulance use; injured regions; and ICD-10–based injury severity score as covariates. Five transfusion ratio points (0.50, 0.75, 1.00, 1.25, and 1.50) were denoted as the knots and 1.00 was designated as the reference point. The area region represents 95% confidence intervals for the estimated adjusted odds ratios.

FFP, fresh frozen plasma; RBC, red blood cell; ICD-10, International Classification of Diseases, 10^th^ Revision

**Figure S6** Non-linear associations between the FFP to RBC ratio or platelet to RBC ratio and adverse events in the sensitivity analyses with restricted cubic spline analysis conducted by altering the definition of massive transfusion to patients who received at least 60 total units of RBC, FFP, and platelets within the first 2 days of admission


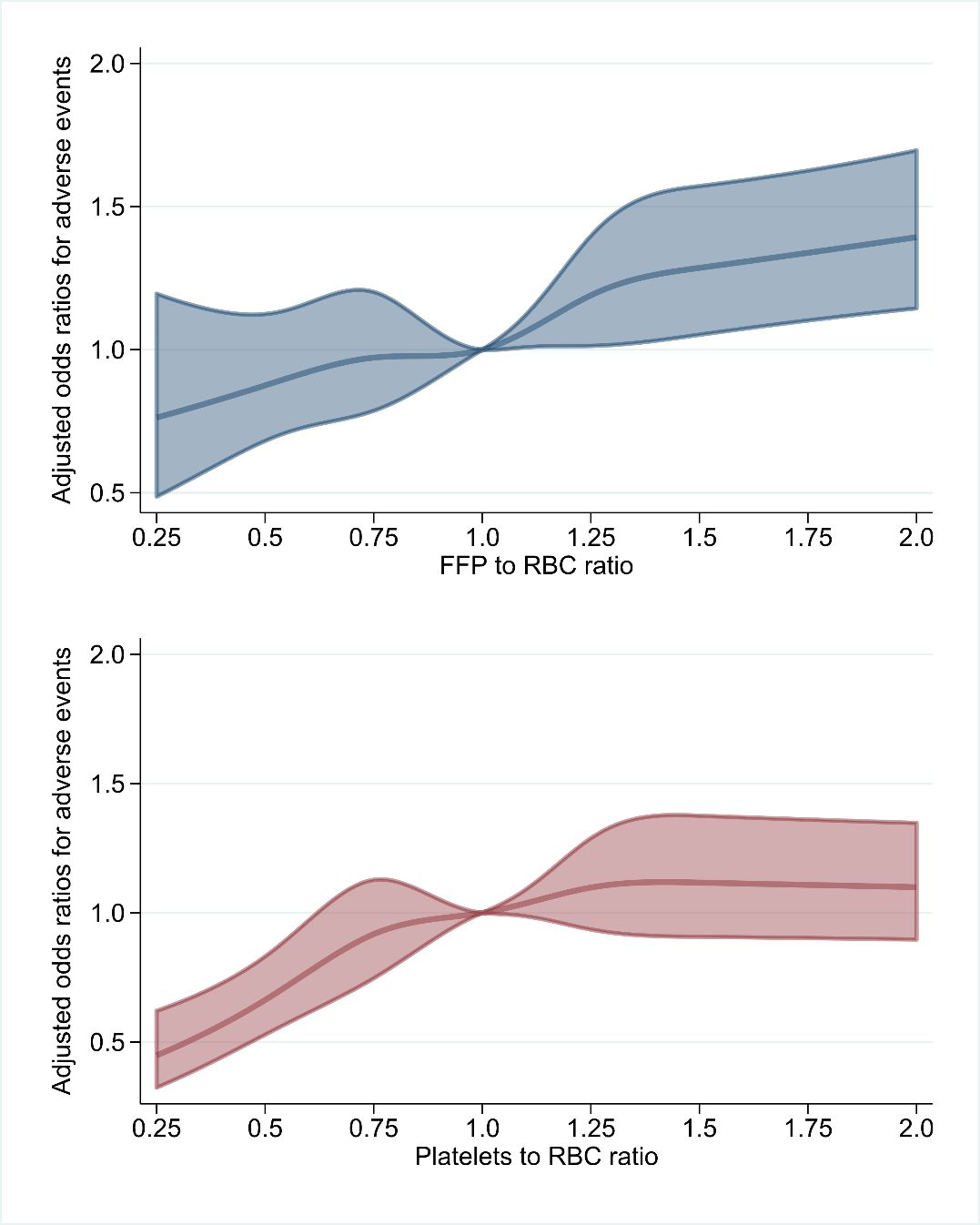


The model was adjusted for calendar year at admission; hospital characteristics; age, sex, and body mass index at admission; Japan Coma Scale at admission; Charlson comorbidity index score; ambulance use; injured regions; and ICD-10–based injury severity score as covariates. Five transfusion ratio points (0.50, 0.75, 1.00, 1.25, and 1.50) were denoted as the knots and 1.00 was designated as the reference category. The area region represents 95% confidence intervals for the estimated adjusted odds ratios.

FFP, fresh frozen plasma; RBC, red blood cell; ICD-10, International Classification of Diseases, 10^th^ Revision

**Figure S7** Non-linear associations between the FFP to RBC ratio or platelet to RBC ratio and in-hospital mortality revealed by sensitivity analyses with restricted cubic spline analysis conducted by restricting the sample to patients admitted to tertiary emergency centers


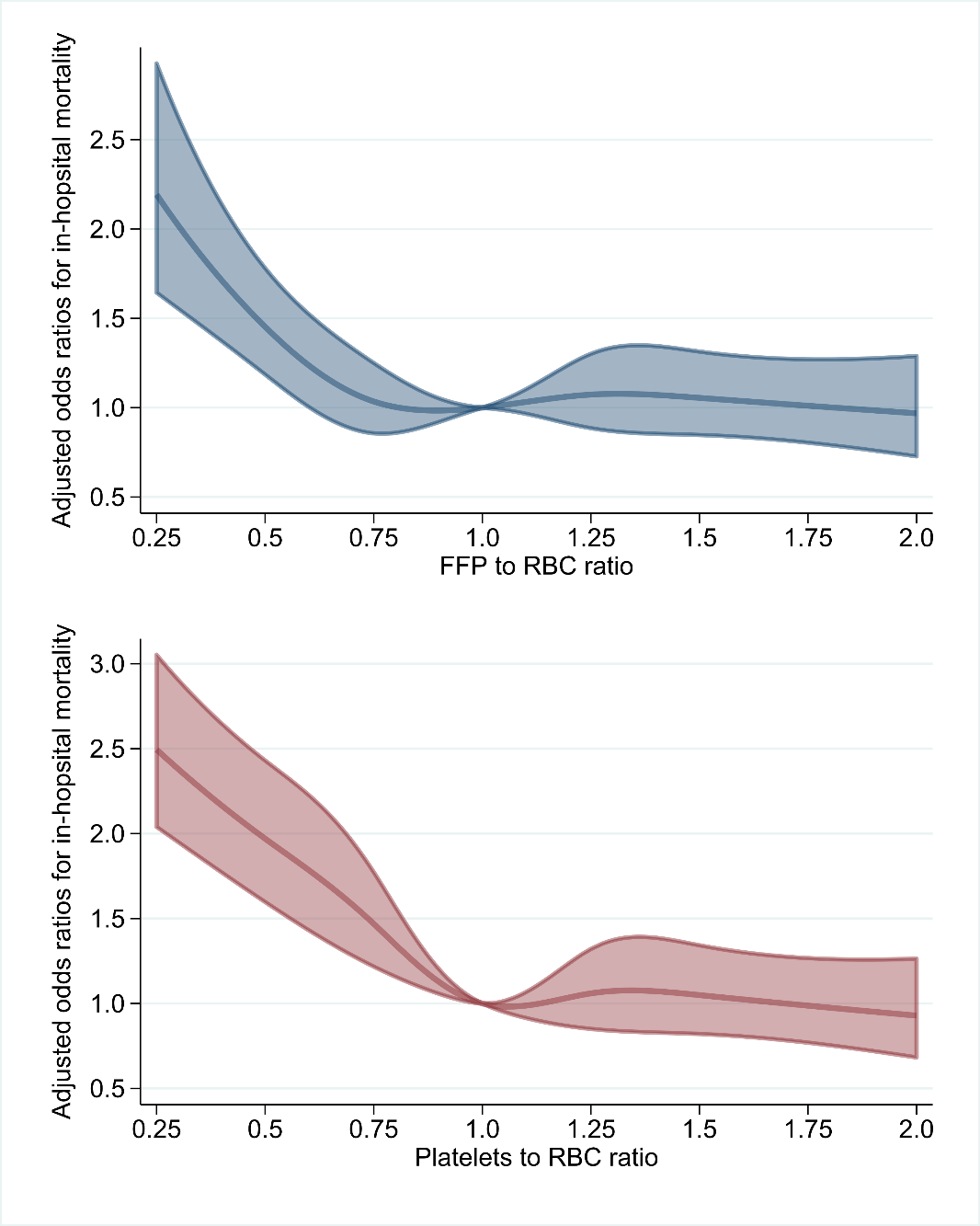


The model was adjusted for calendar year at admission; hospital characteristics; age, sex, and body mass index at admission; Japan Coma Scale at admission; Charlson comorbidity index; ambulance use; injured regions; and ICD-10–based injury severity score as covariates. Five transfusion ratio points (0.50, 0.75, 1.00, 1.25, and 1.50) were denoted as the knots and 1.00 was designated as the reference point. The area region represents 95% confidence intervals for the estimated adjusted odds ratios.

FFP, fresh frozen plasma; RBC, red blood cell; ICD-10, International Classification of Diseases, 10^th^ Revision

**Figure S8** Non-linear associations between the FFP to RBC ratio or platelet to RBC ratio and adverse events in the sensitivity analyses with restricted cubic spline analysis conducted by restricting the sample to patients admitted to tertiary emergency centers


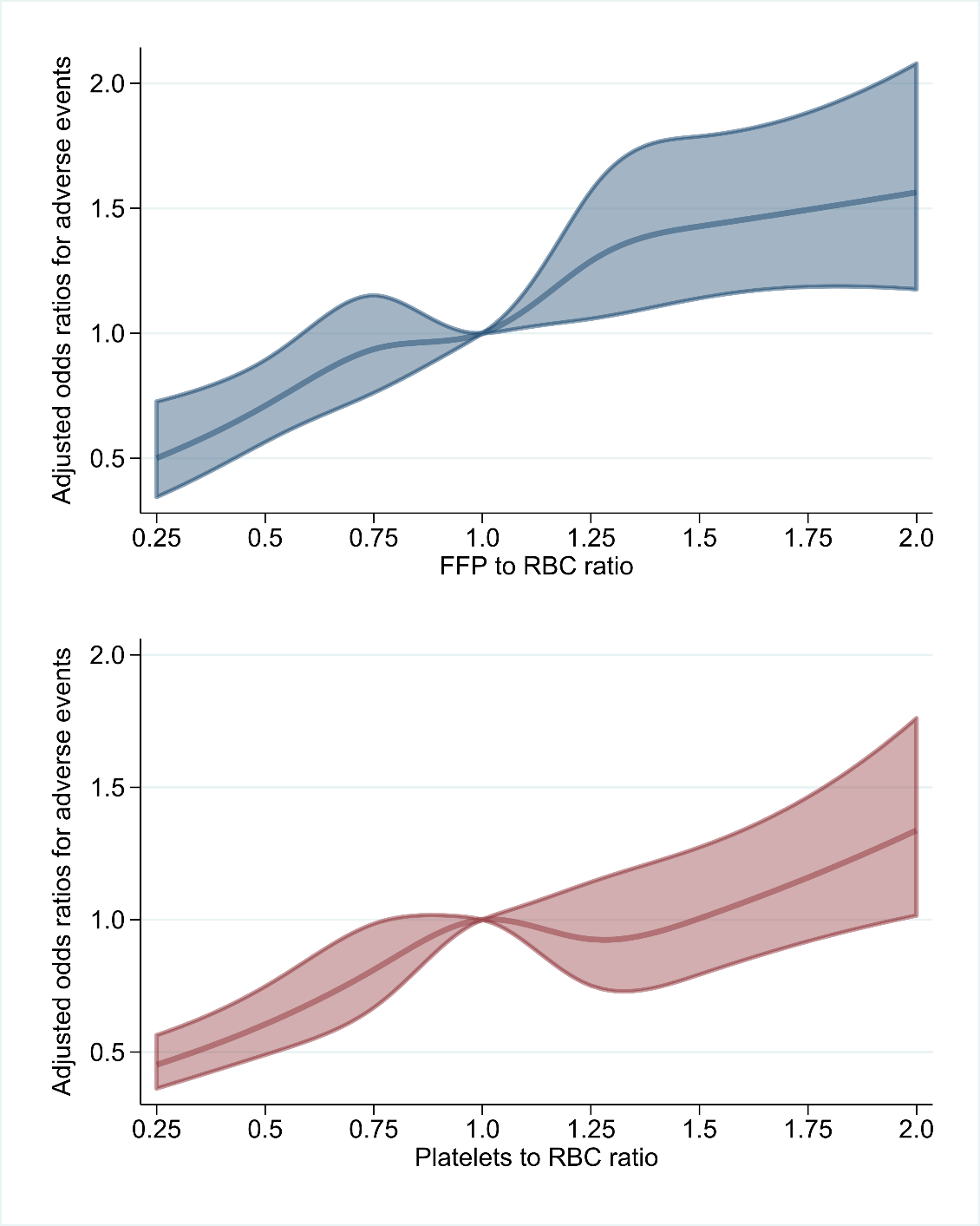


The model was adjusted for calendar year at admission; hospital characteristics; age, sex, and body mass index at admission; Japan Coma Scale at admission; Charlson comorbidity index score; ambulance use; injured regions; and ICD-10–based injury severity score as covariates. Five transfusion ratio points (0.50, 0.75, 1.00, 1.25, and 1.50) were denoted as the knots and 1.00 was designated as the reference category. The area region represents 95% confidence intervals for the estimated adjusted odds ratios.

FFP, fresh frozen plasma; RBC, red blood cell; ICD-10, International Classification of Diseases, 10^th^ Revision

**Figure S9** Non-linear associations between the FFP to RBC ratio or platelet to RBC ratio and in-hospital mortality revealed by sensitivity analyses with restricted cubic spline analysis conducted by restricting the sample to patients who were admitted to hospitals that had continuously provided data to the database from 2011 to 2020


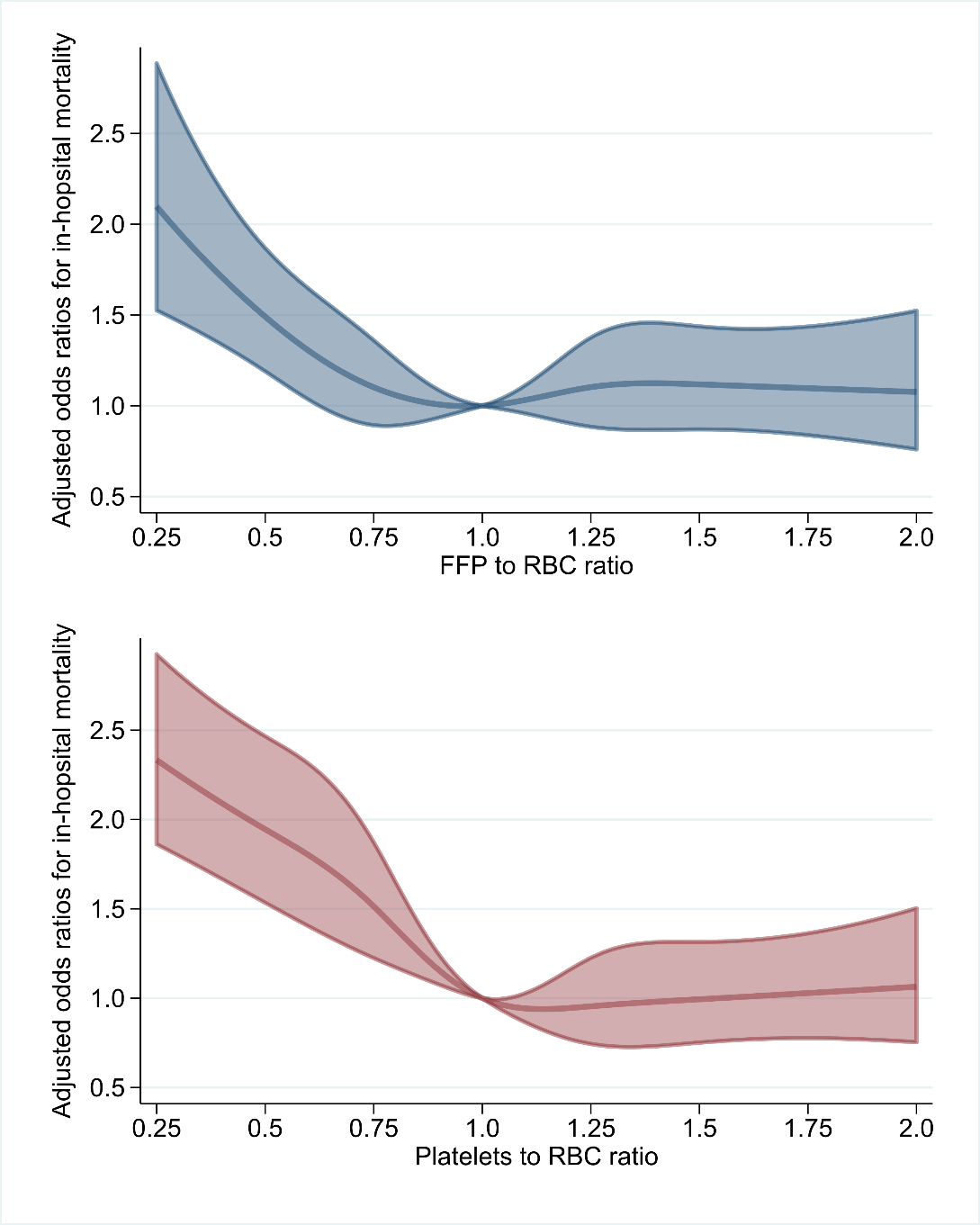


The model was adjusted for calendar year at admission; hospital characteristics; age, sex, and body mass index at admission; Japan Coma Scale at admission; Charlson comorbidity index; ambulance use; injured regions; and ICD-10–based injury severity score as covariates. Five transfusion ratio points (0.50, 0.75, 1.00, 1.25, and 1.50) were denoted as the knots and 1.00 was designated as the reference point. The area region represents 95% confidence intervals for the estimated adjusted odds ratios.

FFP, fresh frozen plasma; RBC, red blood cell; ICD-10, International Classification of Diseases, 10^th^ Revision

**Figure S10** Non-linear associations between the FFP to RBC ratio or platelet to RBC ratio and adverse events in the sensitivity analyses with restricted cubic spline analysis conducted by restricting the sample to patients who were admitted to hospitals that had continuously provided data to the database from 2011 to 2020


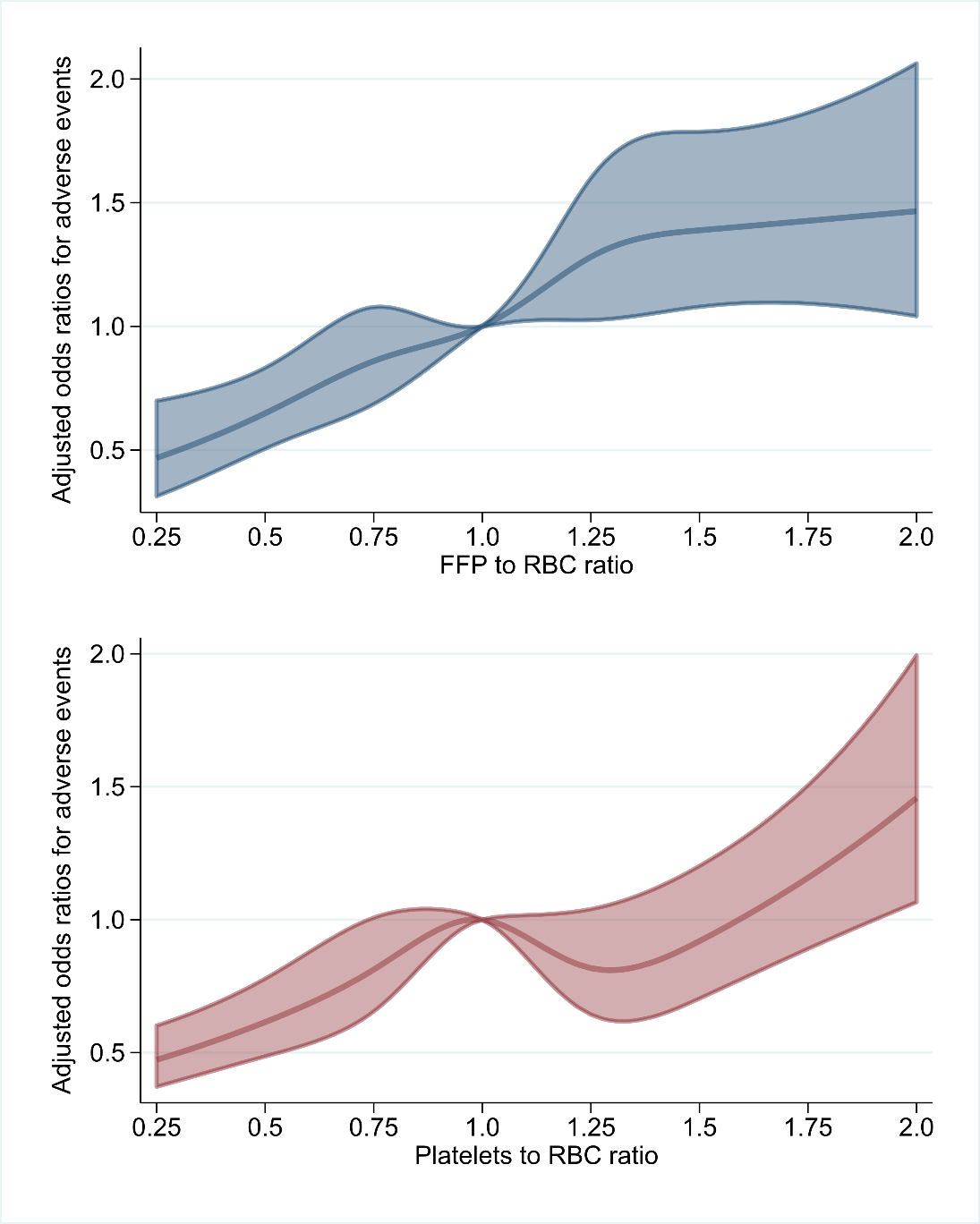


The model was adjusted for calendar year at admission; hospital characteristics; age, sex, and body mass index at admission; Japan Coma Scale at admission; Charlson comorbidity index score; ambulance use; injured regions; and ICD-10–based injury severity score as covariates. Five transfusion ratio points (0.50, 0.75, 1.00, 1.25, and 1.50) were denoted as the knots and 1.00 was designated as the reference category. The area region represents 95% confidence intervals for the estimated adjusted odds ratios.

FFP, fresh frozen plasma; RBC, red blood cell; ICD-10, International Classification of Diseases, 10^th^ Revision
